# Impaired Axonal Transport in Motor Neurons Correlates with Clinical Prion Disease

**DOI:** 10.1371/journal.ppat.1000558

**Published:** 2009-08-21

**Authors:** Vladimir Ermolayev, Toni Cathomen, Julia Merk, Mike Friedrich, Wolfgang Härtig, Gregory S. Harms, Michael A. Klein, Eckhard Flechsig

**Affiliations:** 1 Molecular Microscopy Group, DFG Rudolf Virchow Center for Experimental Biomedicine, University of Würzburg, Würzburg, Germany; 2 Institute of Virology and Immunobiology, University of Würzburg, Würzburg, Germany; 3 Department of Virology, Institute of Infectious Diseases, Charité Medical School, Berlin, Germany; 4 University of Leipzig, Paul Flechsig Institute for Brain Research, Leipzig, Germany; Istituto Superiore di Sanità, Italy

## Abstract

Prion diseases are fatal neurodegenerative disorders causing motor dysfunctions, dementia and neuropathological changes such as spongiosis, astroglyosis and neuronal loss. The chain of events leading to the clinical disease and the role of distinct brain areas are still poorly understood. The role of nervous system integrity and axonal properties in prion pathology are still elusive. There is no evidence of both the functional axonal impairments *in vivo* and their connection with prion disease. We studied the functional axonal impairments in motor neurons at the onset of clinical prion disease using the combination of tracing as a functional assay for axonal transport with immunohistochemistry experiments. Well-established and novel confocal and ultramicroscopy techniques were used to image and quantify labeled neurons. Despite profound differences in the incubation times, 30% to 45% of neurons in the red nucleus of different mouse lines showed axonal transport impairments at the disease onset bilaterally after intracerebral prion inoculation and unilaterally—after inoculation into the right sciatic nerve. Up to 94% of motor cortex neurons also demonstrated transport defects upon analysis by alternative imaging methods. Our data connect axonal transport impairments with disease symptoms for different prion strains and inoculation routes and establish further insight on the development of prion pathology *in vivo*. The alterations in localization of the proteins involved in the retrograde axonal transport allow us to propose a mechanism of transport disruption, which involves Rab7-mediated cargo attachment to the dynein-dynactin pathway. These findings suggest novel targets for therapeutic and diagnostic approaches in the early stages of prion disease.

## Author Summary

For almost a century, prion disease symptoms, such as dementia and motor system defects, have been accompanied with neuropathological hallmarks in the central nervous system. In past decades, discrepancies between neuropathological changes and clinical symptoms showed that the processes triggering the disease remain elusive. We concentrated on the hypothesis that nervous system integrity and axonal properties may play an important role in the disease development. Since motor system defects are typical for prion disease, we investigated the centers of the motor system, red nucleus and hindlimb area of motor cortex. Although intracerebral prion infection led to a 30% to 45% bilateral reduction of labeled neurons in the red nucleus, infection into the right sciatic nerve—the major hindlimb nerve—led to unilateral reduction of labeled neurons in the red nucleus. Up to 94% reduction was found in the neurons of motor cortex hindlimb area. This reduction is probably caused by functional axonal impairments in motor neurons. Prion-induced alterations in protein distribution implicate a mechanism of transport disruption at cargo attachment to the retrograde axonal transport complex. Our work reveals a connection between axonal transport impairments and disease symptoms *in vivo,* providing further insight in the development of prion pathology and suggesting novel targets for therapeutic and diagnostic approaches.

## Introduction

Prion diseases, for example Bovine Spongiform Encephalopathy (BSE) or Creutzfeldt-Jakob disease (CJD), are lethal neurodegenerative disorders caused by the abnormal form (PrP^Sc^) of host prion glycoprotein (PrP^C^). Spongiform vacuolations, accumulation of PrP^Sc^-rich amyloid fibrils, neuronal cell loss, microglial activation and proliferation of astrocytes in the central nervous system (CNS) are typical neuropathological hallmarks [1], which do not always temporally correlate with clinical disease symptoms. Mice bearing one disrupted allele of the PrP gene (*Prnp^0/+^*) develop scrapie symptoms 290 days post intracerebral (i.c.) prion inoculation (dpi), whereas wild-type mice (wt) develop symptoms at 158 dpi. However both mouse lines demonstrate a similar neuropathology already at 140 dpi [2]. In addition, immunodeficient mice show neuropathological changes in the CNS, but develop no clinical symptoms after prion infection [3]. Transgenic mice expressing truncated PrPΔ32-93 (C4/C4) demonstrate no detectable brain pathology upon prion challenge but develop clinical symptoms along with 10–25% neuronal loss in the spinal cord [4].

The study with PrPΔ32-93 mice (C4/C4) implicates that prion-induced impairments and neuronal loss in the spinal cord are sufficient to cause prion disease. The impairments of axon functions such as synaptic degeneration, protein accumulation and changes of microtubule distribution were detected in CJD patients [5],[6], in animal [7] and *in vitro* [8] models of prion disease. However, other studies implied that the transport may not play a key role in prion pathology [9],[10] so that the role of axonal transport still remains elusive.

A growing line of evidence shows that axonal impairments are associated with different neurodegenerative disorders, e.g. Huntington's disease [11]. In Alzheimer's disease (AD), such defects as axonal swellings containing abnormally accumulated proteins, impaired axonal transport with reduced kinesin levels preceded the disease-related pathology in mouse models and in human patients [12].

In this study, we applied axonal tracing as a functional assay for the retrograde transport in five different wild type and transgenic mouse lines. A correlation between impairments in retrograde axonal transport and onset of clinical prion disease was demonstrated independently on incubation time and prion strain establishing a link between molecular changes and clinical symptoms. The combination of prion inoculation into a distinct group of motor neurons with differential localization of proteins involved in the transport processes connects impairments in retrograde axonal transport with prion pathogenesis and suggests molecular mechanism for this process. These findings suggest novel targets for therapeutic and diagnostic approaches in the early stages of prion disease.

## Results

### Axonal tracing of prion-challenged mice

**Figure 1:**
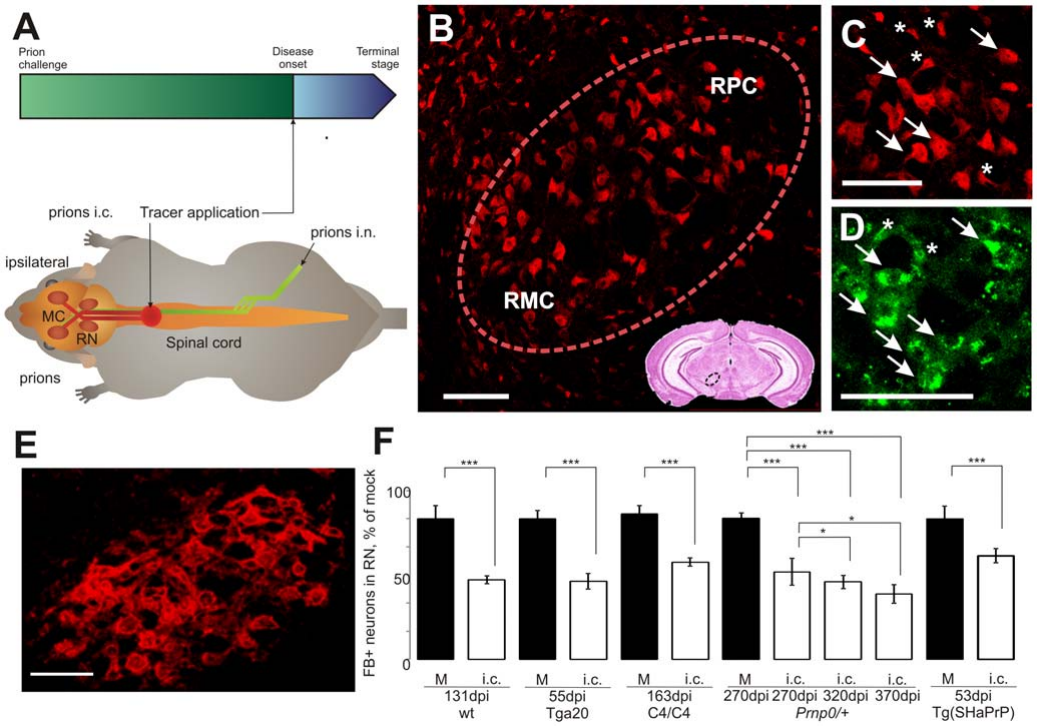
Quantification of retrogradely labeled neurons reveals significant alterations upon prion challenge. (A) Mice were challenged with prions intracerebrally (prions i.c.) or into the right sciatic nerve (prions i.n.). The axonal tracers were applied into the spinal cord before the onset of clinical disease. Retrogradely labeled tracer-positive neurons were quantified in the red nucleus (RN) and motor cortex (MC). (B) The NeuN-positive neurons in the RN (within the dashed line). The RN is a clearly defined center in the midbrain. It is divided into two parts, magnocellular (RMC) and parvicellular (RPC) in the section shown. Small box: anatomic localization of RN on coronal brain section stained with hematoxilin-eosin. (C) Enhanced part of B. The large pyramidal motor neurons projecting via spinal cord were quantified (arrows), but not the smaller ones (asterisks). Scale Bar: 100 µm (D) Fast Blue-positive neurons in the RN. The big pyramidal cells possessed several deposits of FB in the cytoplasm (arrows) and were quantified on z-stacks done on serial cryo-sections. Analysis of z-stacks allowed us to reliably rule out the artifacts (asterisks) from the quantification. Scale bar: 100 µl. (E) *Wisteria floribunda* agglutinin-positive cells are typical for the RN and a proof for the correct localization of tracer-positive neurons. (F) Bilateral reduction of FB-positive (FB+) neurons was observed in the RN of different mouse lines upon intracerebral (i.c.) prion challenge. FB was applied at indicated number of days post prion inoculation (dpi). For the times of disease onset and terminal stage as well as numbers of quantified neurons, see Tables S1 and S2. M – mock, wt – wild type. Unpaired t-test was done using GraphPad Prism software. *** – P<0.0001, * – P<0.02.

The primary objective of our study was to find the molecular mechanisms responsible for the clinical symptoms of prion disease including the motor system defects, such as ataxia and partial hindlimb paralysis [1]. We supposed that the connection between the central nervous system and motor system and, in particular, axonal transport may play a role in prion pathogenesis. Axonal transport was studied in rubro- and corticospinal motor neurons in mouse models for prion disease using retrograde axonal tracing. Wild type (wt) and different transgenic mice were challenged with prions intracerebrally (i.c.) or in the right sciatic nerve (intranervously, i.n.). Prior to the onset of clinical disease, either Fast Blue (FB) [13] or Adeno-Associated Virus expressing DsRed-Express (AAV-REx) was injected into the spinal cord. The tracers were retrogradely transported along axons to label motor neurons of distinct brain regions including the red nucleus (RN) and motor cortex (Figure 1A). Both FB and AAV-REx application did not influence prion disease progression (data not shown). Great care was taken to standardize the quantification of neuron. The anatomical location was verified by staining serial coronal sections with the neuronal marker NeuN (Figure 1B) and co-localization of tracer deposits with NeuN signal. Only large tracer-positive pyramidal motor neurons in the RN were quantified using the analysis of z-stacks done on serial coronal cryo-sections (Figure 1C and D). The proper localization of the RN was also re-verified by staining with *Wisteria floribunda* agglutinin (WFA, Figure 1E), a marker for extracellular proteoglycans at the neuronal surface of RN neurons, but not for neighboring tissue [14],[15].

### Distribution of Fast Blue in the red nucleus following intracerebral prion infection

A bilateral decrease of FB-positive neurons as compared to mock controls was detected in the RN of five different wt and transgenic mouse lines challenged with prions i.c. Despite differences in the incubation times (Table S1), a 43–45% reduction was observed for wt and Tga20 mice. C4/C4 transgenic mice, which demonstrate no typical neuropathology [4] showed a significant 30% decrease of tracer-positive cells in the RN (Figure 1F). Axonal tracing of *Prnp^0/+^* mice was performed at 270 dpi and at 330 dpi immediately after onset of clinical disease (322±2 dpi), and at 370 dpi. A 38% reduction of FB-positive neurons at 270 dpi and a 45% reduction at 330 dpi (similar to wt and Tga20) were counted in the RN. Scrapie-diseased *Prnp^0/+^* animals at 370 dpi demonstrated a 49% decrease of FB-positive neurons in the RN (Figure 1F and Table S1), which indicated a gradual reduction of RN neurons possessing functionally intact projections into the spinal cord during disease development. To clarify whether the decrease of tracer-positive neurons implicating functional impairments in axonal projections is specific for RML prions only, we also applied FB to hamster-adapted transgenic mice Tg(SHaPrP), which were challenged with Sc237 hamster prions. A 43% bilateral decrease of FB-positive cells was observed in the RN (Figure 1F and Table S2) of these mice following i.c. prion infection.

These results demonstrated that despite differences in the incubation times, the bilateral reduction of FB-positive neurons with intact axonal transport correlated with the onset of the clinical symptoms after intracerebral prion challenge. The proportion of affected neurons increased gradually and reached a level of 30–45% at the disease onset independent of the mouse line and prion strain.

### Distribution of Fast Blue in the red nucleus following prion infection in the sciatic nerve

**Figure 2:**
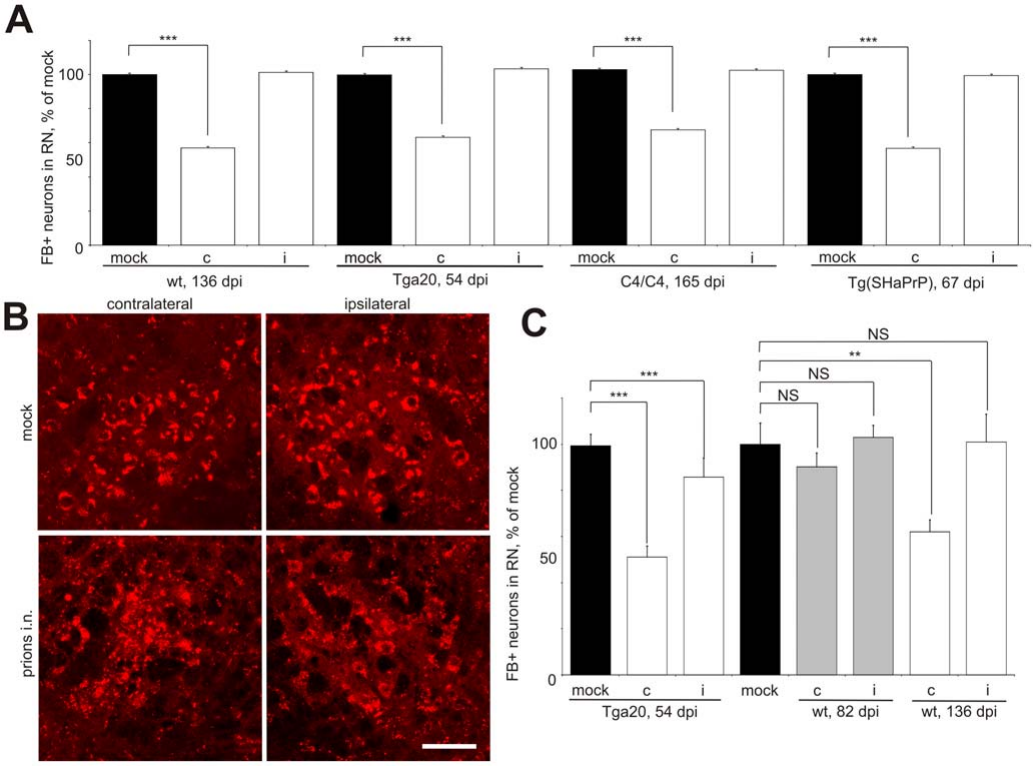
Unilateral decrease of tracer-positive neurons after prion inoculation in the sciatic nerve. Different wild type (wt) and transgenic mouse lines were infected with prions unilaterally in the right sciatic nerve (i.n.) and the tracer was injected in the cervical spinal cord at indicated days post prion challenge (dpi). For the times of disease onset and terminal stages as well as exact numbers of quantified neurons, see Tables S2, S3 and S4. (A) A reduction of Fast Blue-positive neurons (FB+) was demonstrated in the contralateral (c), but not on the ipsilateral (i) RN as compared to the mock controls. (B) The profile of the REx-positive cells differed between contralateral and ipsilateral RN upon i.n. prion inoculation, but not in mock controls. Shown here are the RNs of the Tga20 mice, which injected with AAV-REx at 145 dpi and sacrificed at the terminal stage of the disease. (C) Reduction of REx-positive neurons on contralateral RN was observed in Tga20 and wt mice at the onset of prion disease (54 and 136 dpi after i.n. prion inoculation, respectively), but not at 50% of incubation time (82 dpi). Non-paired t-test was done with GraphPad Prism software. *** – P<0.0001, ** – P = 0.001, * – P = 0.04, NS – non-significant.

In order to assess axonal transport upon selective inoculation into distinct groups of motor neurons, prions were applied into the right sciatic nerve (i.n.). Anatomically, about 90% of RN neurons project to the contralateral side. I.n. inoculation resulted in the initial prion distribution on the contralateral RN followed by bilateral accumulation in the RN, hindlimb motor cortex and thalamus at the terminal stage of the disease [16],[17]. The bilateral prion accumulation upon unilateral i.n. challenge was also recently demonstrated in our lab for the RN and MC at both onset and terminal stage of clinical disease [18], which is in agreement with the data on i.n. inoculations from other groups [16],[17]. Moreover, similar neuropathological features, such as spongiosis and activation of astrocytes were also observed in the RN bilaterally (Figure S1). Surprisingly, upon application of FB shortly before the onset of disease symptoms, a 43% and 37% reduction of FB-positive cells was found exclusively on the contralateral RN side in wt and Tga20 mice, respectively. C4/C4 transgenic mice showed a 33% unilateral decrease of FB-positive neurons, which correlated with the unilateral reduction for wild-type (wt) and Tga20 animals. A 28% unilateral decrease of FB-positive cells was also observed in the RN of Tg(SHaPrP) mice upon i.n. challenge with hamster prions (Figure 2A and Tables S2 and S3).

In order to understand whether prion challenge affects only the retrograde transport mechanism utilized by FB, we exactly reproduced the tracing experiment on i.n. challenged wt and Tga20 mice using a viral vector based on adeno-associated virus [19],[20],[21] expressing the fluorescent marker DsRed-Express (REx). The numbers of FB- and REx-labeled neurons did not significantly differ for the mock controls. Moreover, REx and FB co-localized in the RN neuronal cells after injection into the spinal cords of the same animals (data not shown). Similar to FB, the difference between contra- and ipsilateral sides upon i.n. prion challenge could be clearly seen on the same z-stacks done on RN section of i.n. challenged mice but not of mock controls (Figure 2B). The quantification of REx-positive neurons for wt and Tga20 mice revealed a 39% and 49% decrease of tracer-positive neurons on the contralateral side of the RN, respectively (Figure 2C and Table S4). The reduction of tracer-positive neurons initially demonstrated for FB was confirmed for AAV-REx. The tracing experiment at 50% of incubation time (at 82 dpi upon i.n. challenge) demonstrated no significant differences of labeled RN neurons with mock controls, which reveals the important role of this center in the development of the disease and supports the view that appearance of the axonal transport impairments in the RN neurons is connected with the disease onset.

**Figure 3:**
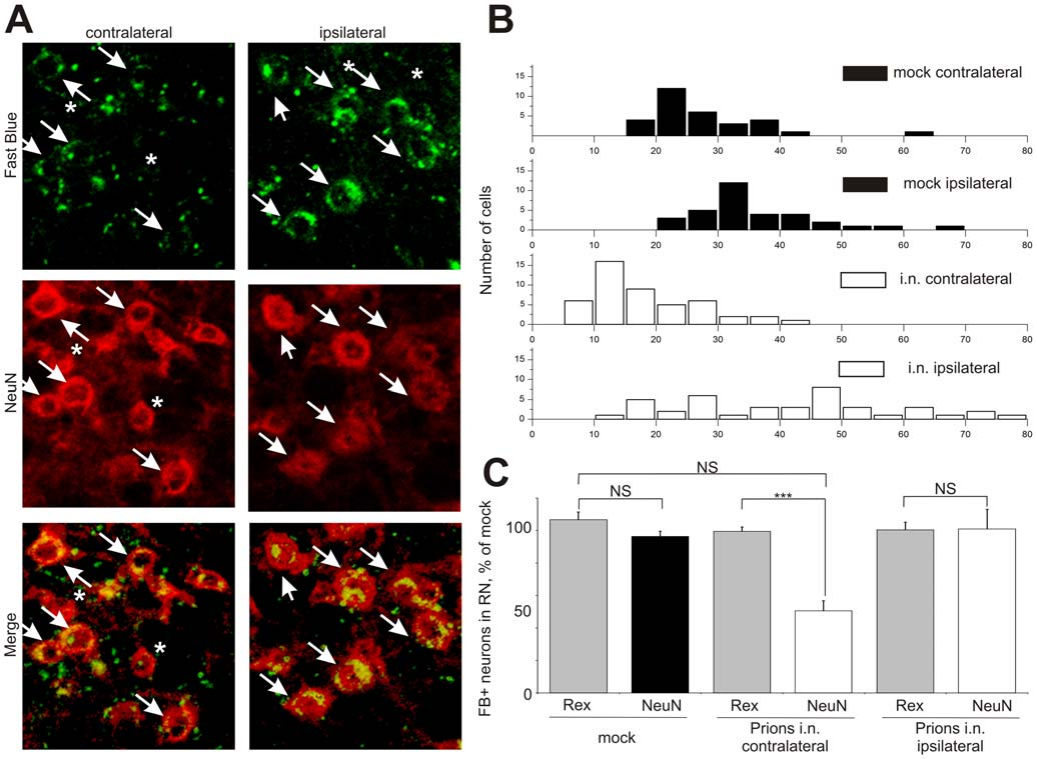
Reduction of tracer-positive cells in the red nucleus is not attributed to neuronal loss. (A) Co-localization of FB (green) and NeuN (red) analysis in the contralateral and ipsilateral RN of mice upon prion challenge in the sciatic nerve (i.n.) done at the onset of clinical disease (152 dpi). A population of viable NeuN-positive cells is FB-negative (asterisks), which implicates the defects in axonal transport. In other cells NeuN and FB co-localize (arrows, yellow). Scale bars: 50 µm. (B) Distribution histogram of the relative FB to NeuN fluorescence analyzed on z-stacks in the Regions of Interest (ROI). The analysis quantitatively demonstrates that FB fluorescence co-localizes with NeuN in mock controls. 44% of contralateral neurons was below the background threshold and considered FB-negative, and 7%—on the ipsilateral RN. (C) Quantification of NeuN-positive neurons in the sampled mock and i.n. mice revealed no differences between mock and inoculated animals despite the reduction of REx-positive cells. Unpaired t-test was done using GraphPad Prism software. *** – P<0.0001. NS – non-significant.

Our system combining unilateral inoculation of prions into the right sciatic nerve and axonal tracer application enabled investigation of axonal defects specifically connected with prion pathogenesis. The ipsilateral side of the RN did not show any significant decrease of tracer-positive neurons and could be used as an internal control. The differences between the ipsilateral site and the contralateral – initially targeted with prions were directly observed on the same z-stack done on serial cryo-sections of i.n. challenged mice but were not detectable in the mock controls. Moreover, on the contralateral side of the RN, we observed a fraction of NeuN-positive neurons, which did not contain FB (Figure 3A, asterisks). In contrast, the ipsilateral RN did not contain such cells but only the cells containing both FB and NeuN (Figure 3A, arrows; for overview, see Figure S2). This observation was confirmed on both visual and computerized co-localization analysis. Due to high background we still observed the fluorescent objects in the vicinity of NeuN-positive cells on the contralateral RN, which were not perfectly co-localizing. In order to perform systematic stereological analysis, we defined Regions of Interest (ROI) according to the NeuN-positive fluorescence in several mock and i.n. samples (Figure S3A) and quantified the FB fluorescence as a per cent of the NeuN fluorescence in z-stacks using available software tools. The analysis of more than 150 cells from 3 i.n. and 4 mock samples revealed practically all the cells in mock controls to contain FB fluorescence over 18% to the level of NeuN signal, which was over the calculated background value of 17%. Fourty-four per cent of the cells in the contralateral RN (21 of 47 totally analyzed) demonstrated the relative FB fluorescence under the background level, and only 7% (3 of 40 analyzed) – in the ipsilateral RN (Figure 3B).

To further understand whether the reduction of the tracer-positive neurons can be explained by the neuropathological changes leading to the neuronal disruption, we quantified the NeuN-positive cells in the RNs of sampled mock (n = 3) and i.n. challenged (n = 4) mice. We did not observe significant differences in NeuN-positive cells between mock and i.n. inoculated mice despite very clear difference in REx-positive cells (Figure 3C and Table S5). Taken together, this data implies that the decrease of tracer positive cells upon prion challenge cannot be simply explained by neuronal loss but by the impairments in the delivery of transported cargo – the FB molecules or AAV-REx.

**Figure 4:**
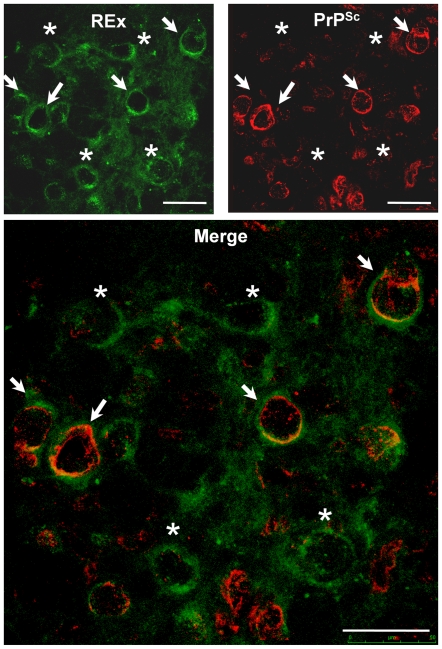
Co-localization analysis of REx and PrP^Sc^. Co-localization analysis of REx (green) and PrP^Sc^ (red, stained with ICSM35 antibodies) in the RN of wt mice was done at 152 dpi upon i.n. prion inoculation. In a certain cell population of REx and PrP^Sc^ co-localize (arrows, yellow). Other cells are REx-positive but do not contain PrP^Sc^ (asterisks). Scale bars: 50 µm.

The dramatic reduction of tracer-positive neurons indicated functional impairments of retrograde transport in the RN neurons. These impairments occurred bilaterally upon i.c. and unilaterally – upon i.n. prion challenge. However, the accumulation of PrP^Sc^ and neuropathological changes were observed in the RN bilaterally also upon i.n. inoculation [16],[18]. In order to clarify whether the decrease of tracer-positive neurons directly reflected the fraction of the PrP^Sc^-containing neuronal perikarya, we performed co-localization experiment and analysis of PrP^Sc^ and REx in the RN of i.n. prion-challenged wt mice. The fraction of the REx-positive cells co-localized with PrP^Sc^ at the onset of clinical prion disease (152 dpi, Figure 4, arrows), but the rest of REx-positive cells was PrP^Sc^-negative (Figure 4, asterisks). This experiment implies that PrP^Sc^ possibly does not switch off the axonal transport in a trigger-like manner upon arrival in the somas of RN neurons, but likely initiates a gradual decrease of transport functions in axons during the neuroinvasion process. This assumption is also supported by the gradual decrease of tracer-positive neurons in *Prnp^0/+^* mice upon i.c. prion challenge (Figure 1F).

### Reduction of DsRed-Express-positive neurons in the motor cortex

**Figure 5:**
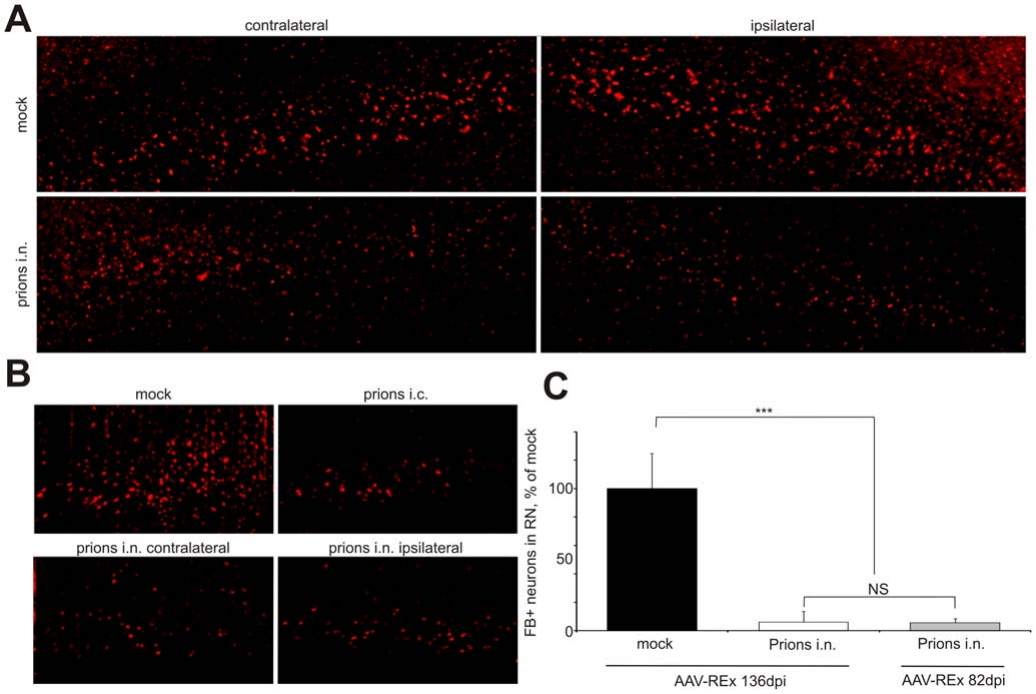
Reduction of DsRed-Express-positive neurons in motor cortex. (A and B) Bilateral reduction of REx-positive neurons in the motor cortex (MC) of wild type (wt) mice upon prion challenge into the right sciatic nerve (prions i.n.) or intracerebrally (prions i.c.), as compared to mock controls, shown with confocal microscopy (A) and ultramicroscopy (B). (C) Quantification of REx-positive neurons on confocal images of MC shows a 94±3% (n = 4) reduction of tracer-positive cells in the MC of wt mice inoculated with prions i.n. at the disease onset (AAV-REx application at 136 days post prion inoculation, dpi) and a 95±1% reduction (n = 3) at 50% of incubation time (AAV-REx application at 82 dpi). Unpaired t-test was done using GraphPad Prism software. *** – P<0.0001. NS – non-significant. Scale bars: 100 µm.

Inoculation into the right sciatic nerve results in a bilateral prion distribution in the MC, the brain center responsible for voluntary movements in rodents and humans. To assess retrograde axonal transport in this area, we compared REx-positive neurons in MC of mock- and prion-inoculated mice using confocal microscopy and our custom-built ultramicroscopy system [18]. Significant differences in REx-positive cells were not detected in wt mock controls, but the difference to prion-challenged animals was clearly visible (Figure 5A). Ultramicroscopy is a method significantly differing from confocal imaging. It requires no cryo-sectioning and allows for imaging of the large region in the MC. Similar to confocal microscopy, ultramicroscopy revealed clearly visible reduction of REx-positive neurons in the MC of wt mice inoculated with prions i.n. as well as i.c. as compared to the mock controls (Figure 5B and [18]). The quantification of the REx-positive neurons in the hindlimb area of the MC was done on combined confocal images.

Extra precautions were taken to assure accurate and standardized area localization as well as reliable and uniform quantification and analysis of labeled cells. The confocal stacks were simultaneously contrasted and thresholded, and the REx-positive cells were quantified within the same combined stack. This approach ruled out the possible artifacts between mock and infected samples due to different contrasting or thresholding. The quantification procedure was normalized according to the REx deposit properties assessed by co-localization with the NeuN neuronal marker as was demonstrated earlier [18]. A remarkable 94±3% bilateral reduction of REx-positive neurons was found in the MC of i.n. challenged wt mice (n = 4). Surprisingly, the tracing experiment with wt animals at 50% of incubation time (82 dpi) demonstrated the reduction of the tracer-positive neurons (95±1%, n = 3) similar to the one obtained immediately prior to the onset of the clinical disease (Figure 5C). Previously, we already demonstrated that both methods of confocal and ultramicroscopy revealed quite similar reduction of tracer-positive neurons in the same MC regions at the onset of prion disease [18]. Moreover, we also showed that the number of NeuN-positive neurons was not affected at the onset of clinical prion disease upon i.n. challenge in the MC [18]. This experiment rules out the possibility that the massive reduction of tracer-positive cells can be attributed to the neuronal loss upon prion challenge.

### Alternative hypotheses for labeled neuron decrease

The decrease of tracer positive neurons implies impairments of axonal transport caused by the prion infection. However, there are alternative explanations of observed phenomenon. The possibility that the observed reduction of tracer positive cells can be explained by the reduction of neurons in the RN and MC seems not very plausible because of comparable number of NeuN positive neurons in the RN (Figure 3A) and MC [18]. A further hypothesis implies that the observed decrease could be attributed to the altered tracer uptake or altered number of axons in the cervical spinal cord at the injection site. Earlier we demonstrated that ultramicroscopy z-stacks of REx-positive neurons in the cervical spinal cord immediately cranially to the tracer injection site do not differ between mock controls and i.n. infected mice [18]. In order to demonstrate, whether the axons in the spinal cord white matter are altered upon prion challenge, we performed Toluidine Blue staining. Toluidine Blue stains myelin sheath visualizing neuronal projections in the spinal cord. The Toluidine blue staining showed no significant differences between spinal cord samples of mock and prion-infected mice. Some axonal swellings were observed in the spinal cord and represent the variable axon caliber resembling to our knowledge the typical appearance of myelinated and non-myelinated axons. Furthermore, since these swellings appear not only in the spinal cords of prion-infected, but also of the mock animals, and since they are not restricted to the rubro- or corticospinal tracts, we assume that they probably could be partially attributed to experimental artifacts, for example as a result of tracer injection. A certain degree of axonal degeneration upon prion infection, however, cannot be absolutely excluded with these experiments.

### Altered distribution of marker proteins upon prion challenge

Further, we aimed to clarify whether the transport impairments documented by the altered delivery of the retrograde cargo – the FB or AAV-REx – can be characterized by the changes in protein distribution. The localization of various proteins either previously connected with prion pathology or involved in the retrograde transport processes was analyzed using immunohistochemistry.

**Figure 6:**
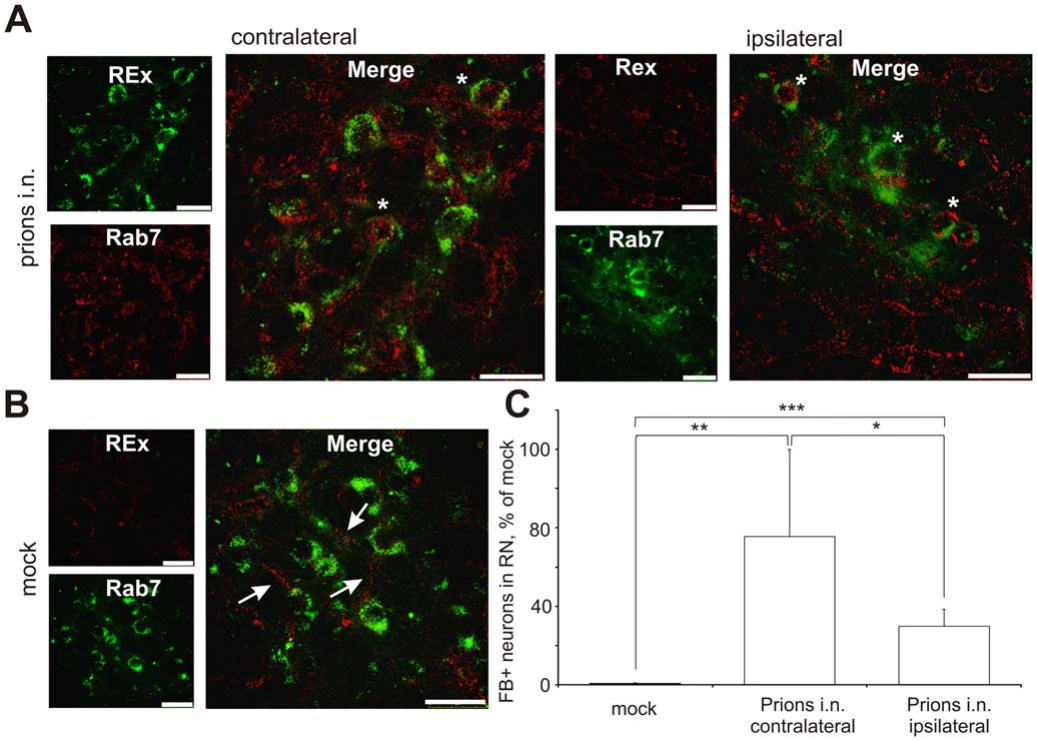
Co-localization analysis of Rab7 and REx in the red nucleus of wild type mice. Co-localization analysis of Rab7 (red) and REx (green) was performed in the red nucleus (RN) of wild type (wt) mice upon i.n. prion challenge (A) or in mock controls (B). Rab7 in mock controls was localized mostly in the neuronal projections (arrows), but not in the vicinity of RN neuronal somas. Upon prion challenge Rab7 localization was more diffuse. Rab7 was also localized in the vicinity of REx deposits within the same cells (asterisks). (C) Analysis of Rab 7 and REx fluorescence within the regions of interest (ROI) defined in the contralateral and ipsilateral RN of animals infected with prions i.n. or in mock controls. Unpaired t-test was done using GraphPad Prism software. *** – P = 0.0003, ** – P = 0.0077, * – P = 0.0460. Scale bars: 50 µm.

AAV enter the cells via clathrin-mediated endocytosis upon binding to the heparan sulfate proteoglycan. After entering the neuronal cell, AAV particles are internalized into Rab7-positive late endosomes. Rab7 mediates the endosome binding to the dynein-dynactin complex via the p150^GLUED^ protein and are retrogradely transported towards neuronal bodies [22]. We analyzed the distribution pattern for Rab7, a protein responsible for recruitment of late endosomes into the retrograde transport pathway. In the RN of i.n. prion-challenged animals at the onset of prion disease (at 152 dpi) the pattern of Rab7 distribution was diffuse, sometimes in the close vicinity or within the REx-positive cells (Figure 6A). In the mock controls Rab7 immunoreactivity concentrated mostly in the neuronal projections not co-localizing with REx-positive neurons (Figure 6B). The analysis of prion infected mice demonstrate that Rab7 did not directly co-localize with REx but was located in the vicinity of it in the RN cells, but not of mock controls. We performed analysis of relative fluorescence of Rab7 on confocal z-stacks in the ROIs defined according to the REx-positive neurons (Figure S3B). The Rab7 fluorescence was quantified as per cent to the REx signal in the same cell volume. The Rab7 fluorescence was not present in the REX-positive cells of mock controls (0.7±0.3% of REx fluorescence intensity). The REx-positive cells of i.n. infected mice did contain a lot of Rab7 signal, in ipsilateral RN (29.8±8.9%) and especially in contralateral RN cells (75.4±44.6%) (Figure 6C).

The staining for the p150^GLUED^ protein involved in the binding of late endosomes to the dynein-dynactin transport complex demonstrated a bilateral decrease of immunoreactivity in the RN of i.n. prion-challenged mice as compared to the mock controls (Figure S4).

**Figure 7:**
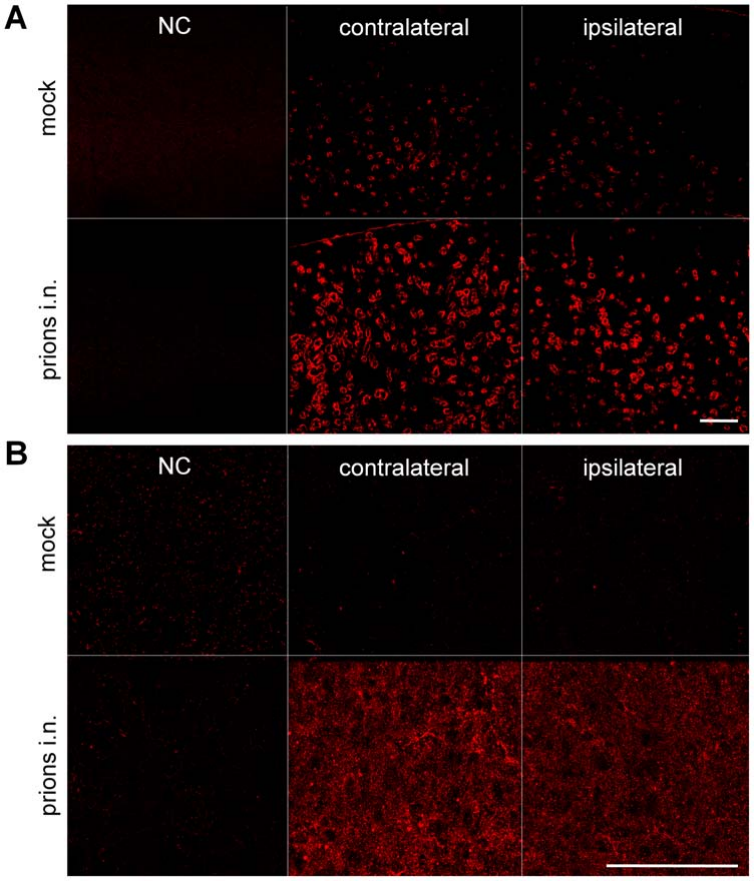
Altered protein expression in motor cortex upon prion challenge into the sciatic nerve. The wild type mice were analyzed at the time point before the onset of clinical disease (145 dpi) upon prion challenge into the sciatic nerve. (A) Bilaterally, a 2.5-fold enhanced ubiquitin expression was detected in motor cortex (MC) of prion-challenged mice as compared to mock control. NC – negative control with omitted primary antibodies. (B) Bilaterally, a 6-fold enhanced Notch expression was detected in the MC of prion-challenged mice as compared to mock control. NC – negative control with omitted primary antibodies. Scale bars: 100 µm.

Highly aggregated PrP^Sc^ deposits were shown to be ubiquitinated in axons as well as neuronal bodies of CJD patients [6]. Analysis of MC cryo-sections stained with anti-ubiquitin antibodies revealed 2.5-fold enhanced ubiquitination at the onset of clinical disease (152 dpi) after i.n. prion challenge as compared to the mock controls (Figure 7A). This data indicated induced protein degradation processes in the PrP^Sc^-containing hindlimb area of MC, which also contained neurons with axonal transport defects.

Activation of different forms of Notch protein (Notch-1, Notch-2 etc.) inhibits axonal growth during neuronal development as well as elaboration and maintenance of mature axons [23],[24]. Increased levels of the Notch-1 intracellular domain (NICD) were also detected in the neurons of prion-infected mice [25]. Upon analysis of the MC cryo-sections stained with anti-Notch-1 antibodies we found 6-fold elevated levels of NICD at the onset of clinical disease (152 dpi) in the MC of i.n. inoculated animals as compared to the mock controls (Figure 7B), which indicated possible disruption of axonal maintenance upon prion challenge.

The staining for dynamitin, a component of the dynein-dynactin transport protein complex, however, did not reveal any differences between prion-challenged and mock mice (Figure S5). This data confirms the previous observation that mice bearing mutation in the cytoplasmic dynein do not show any differences in prion disease incubation time nor neuropathology as compared with wild type littermates [9].

## Discussion

Axonal transport is a process important for neuronal viability and differentiation. It is often impaired during development of various neurodegenerative diseases, such as AD or amyotrophic lateral sclerosis [26]. Several experimental reports performed with animal models of prion disease [7],[8] or based on the analysis of CJD patients [5],[6],[27],[28] implied general axonal defects, such as axonal swellings or altered distribution of transport-related proteins possibly contributing to prion pathogenesis, but did not establish a correlation between axonal impairments and clinical symptoms, nor demonstrated any functional axonal impairments *in vivo*. Other studies, however, suggested no correlation between axonal transport and prion disease [9],[10].

The RN is a part of the extrapyramidal motor system, along with cerebellum, substantia nigra and other brain centers as well as rubrospinal tract. A great majority of the extrapyramidal neurons project via the rubrospinal tract on the contralateral side. The MC belongs to the pyramidal motor system, which innervate the muscles via the brainstem or spinal cord. The neurons in the RN are connected with the MC and also interconnected between the right and left sides. Despite the complexity of the neuronal connections we detected the reduction of the tracer positive neurons bilaterally upon the i.c. prion infection, but unilaterally in the RN of mice unilaterally challenged in the sciatic nerve. This reduction also seemed to correlate well with the appearance of clinical symptoms. The clinical symptoms in the Tga20 mice, such as hindlimb paralysis, had clear unilateral character. The other mouse lines, however, showed much more balanced motor system defects. Such reductions were observed at the onset of clinical prion disease in five different mouse lines despite very different incubation times (59±1 to 322±2 dpi for Tga20 and *Prnp^0/+^* mice, respectively). The observed defects were independent on prion strain and inoculation route (Figures 1F, 2A, 2B). The reduction of tracer-positive neurons could not be explained simply by the loss of neuronal cells. We observed a fraction of NeuN-positive neurons in the RN to be FB-negative implying the axonal transport impairments in viable neurons (Figure 3A). Moreover, the quantification of NeuN-positive neurons revealed no difference between prion-infected and mock mice neither in the RN (Figure 3C) nor in the MC [18]. The reduction of tracer-positive neurons very likely implies the functional impairments of the axonal transport in the living cells. An alternative hypothesis, which suggests alterations in the tracer uptake, deposition or, in the case of AAV-Rex, expression could be ruled out since differences in spinal cord REx profiles between i.n. infected and mock mice could not be observed [18]. Prion-induced changes in the axons could also make further possible input in a reduction of labeled neurons. The Toluidine Blue staining of axonal myelin in the spinal cord did not demonstrate significant differences between mock controls and i.n. challenged mice. We observed certain axonal swellings, which can be partially attributed to the experimental artifacts (Figure S6). Despite a certain degree of axonal degeneration could not be absolutely ruled out, it cannot explain prion-induced reduction of the labeled neurons. Moreover, since the tracer-positive neuron profile in the RN upon intramuscular AAV-REx application (Ermolayev et al., unpublished results) is not different from the results upon the AAV-REx application in the cervical spinal cord, we assume that axonal degeneration probably does not play a critical role in prion pathogenesis.

We believe that the observed reduction of tracer-positive neurons in the RN and MC could be most probably explained by the functional impairments in axonal transport. Since the application of two different tracers revealed a very similar reduction of tracer-positive cells in the RN of different mouse lines, we find that impairments of axonal transport belong to a general phenomenon and are not limited to a certain type of neuronal cells or represent a restricted subset of retrograde transport pathways.

PrP^Sc^ probably initiates a process of gradual axonal transport disruption, which could be demonstrated by the gradual decrease of FB-positive neurons in *Prnp^0/+^* heterozygous mice (Figure 1F) as well as from the observation that PrP^Sc^ partially colocalizes with the tracer-positive cells in the RN (Figure 4).

We found altered localization of Rab7 and p150^GLUED^, proteins involved in retrograde axonal transport (Figure 6 and Figure S4, respectively) in the RN of prion-challenged mice. Since the dynamitin localization remains unaffected (Figure S5), it could be assumed that the dynein-dynactin transport complex itself is not affected during prion pathogenesis, which is consistent with previously reported data [9]. Moreover, since map-2 and tau distribution remains unaffected in prion-infected as compared to mock animals (data not shown) as well, it could be assumed that the microtubule network and dynein-dynactin retrograde transport complex remain unaffected. The attachment of the cargo to this complex, however, may represent a target of early prion pathogenesis, which is supported by the altered distribution of Rab7 and p150^GLUED^ proteins, which are either involved in the recruitment of the vesicles into the dynein-dynacting pathway [26], or directly involved in the attachment of the cargo to it [22]. The p150^GLUED^ distribution is bilaterally diminished. The pattern of Rab7 immunoreactivity is altered bilaterally. However, the quantitative stereological analysis reveals that the neurons in the contralateral RN show 2.6-fold more Rab7 in the neuronal bodies at the onset of clinical prion disease. These data point on the important role of small Rab7 protein in the development of prion-induced pathological changes.

The cells in the hindlimb area of MC demonstrated enhanced protein degradation in prion-challenged mice at the onset of clinical disease, which was indicated by elevated ubiquitin level (Figure 7A). The bilateral 6-fold activated Notch-1 expression in the hinlimb area of MC also at the onset of the disease (Figure 7B) suggested impairments in the axonal maintenance as a component of prion pathogenesis. Along with elevation in the experimental model of prion disease [25], Notch expression was reported to be up regulated also in the brains of AD patients [29]. AD is a neurodegenerative disease, which was recently also connected with axonal dysfunctions [12].

The tracing experiment performed at 82 dpi (50% of incubation time upon i.n. prion challenge) did not reveal the differences of REx-positive neurons in the RN between prion-infected animals and mock controls (Figure 2C). Surprisingly, in the MC of the same mice we detected as many impaired neurons as in the animals immediately before the onset of the clinical disease (Figure 5C). Similar proportions of damaged cortical motor neurons at the onset of the disease and at 50% of incubation time demonstrate that dramatic alterations in the hindlimb area of the MC can still be compensated. The RN along with other brain centers in rodents such as cerebellum is responsible for muscle coordination during the movements. Since axonal transport defects in RN neurons appear at the onset of clinical disease, but not at the 50% of incubation time, we assume that this center plays a critical role in the development of clinical symptoms. Possibly, the RN plays a role of so-called “clinical target areas”, the regions required for the clinical disease development [30]. The other brain centers involved in the movement coordination may also play a role in prion pathogenesis, but their study was not the goal of this report. The preferential role of certain brain areas was also observed for other neurodegenerative diseases, for example certain population of cholinergic neurons in AD [31].

The present study shows that the reduction of labeled neurons in the RN and MC are caused by the prion-induced impairments of retrograde transport in the axons projecting via spinal cord. Such impairments in the RN neurons temporally correlate with clinical symptoms of the disease, such as ataxia and hindlimb paralysis, in five different mouse lines and different prion strains independently on the inoculation route. We postulate a crucial role of certain brain centers, e.g. RN, in prion pathogenesis. The marker protein distribution suggests that the these impairments may occur not due to the disruption of the microtubule network or the dynein-dynactin transport complex, but probably due to the alterations in the cargo attachment to the transport machinery. We suggest a further pathogenic mechanism in prion disease initially involving functional disruption of axonal transport in rubrospinal motor neurons already on the early stages of prion neuroinvasion. These results suggest novel targets for diagnostic and therapeutic approaches, which could be directed at the axonal transport processes with the focus on the late endosome attachment to the retrograde transport pathway. Further studies would allow better characterization of molecular mechanisms underlying the axonal transport disruption at the prion pathogenesis as well as distinct similarities with other neurodegenerative disorders.

## Materials and Methods

### Mouse lines used in the study

C57Bl/6 or wild type (wt), Elevage Janvier, Le Genest Saint Isle, France. Tga20, expresses approximately 10-fold PrP as compared to the wild type under control of PrP promoter on PrP knock-out genetic background [32]. Prnp^0/+^, possesses one intact and one disrupted allele of PrP gene [2]. C4/C4 express truncated Δ32-93PrP on PrP knock-out genetic background [4]. TG(SHaPrP), expresses approximately 20-fold Syrian hamster PrP as compared to the wild type hamster under control of PrP promoter on PrP knock-out genetic background [33].

### Mouse inoculation and tracer application

All procedures with laboratory animals were approved by the committee for the Care and Use of Laboratory Animals of the Government of Bavaria. Either 30 µl inoculum was injected intracerebrally (i.c.) or 1 µl inoculum – into right sciatic nerve (i.n.). 1% RML (1% brain homogenate of terminally scrapie-sick CD1 mice infected with the Rocky Mountain Laboratory scrapie strain) was used as mouse prion inoculum, or 1% Sc237 as a hamster inoculum. 1% brain homogenate from healthy littermates was used for mock controls. Inoculations into the sciatic nerve were performed as previously shown [16] with minor changes. Briefly, animals were anaesthetized with 40 mg/kg Ketanest (Parke-Davis, c/o Pfizer, Freiburg, Germany) and 40 mg/kg Rompun (Bayer, Leverkusen, Germany). The right sciatic nerve was surgically exposed by dislodging M. gluteus superficialis and M. biceps femoris, placed onto a metal plate (2×5×1 mm) and 1 µl of 1% RML was injected into the nerve with a 33-gauge Hamilton syringe over a period of 5 min. Great care was taken to visually control and assure accurate inoculation into the nerve. After inoculation, the sciatic nerve was repositioned and the lesion was closed with Vicryl resorbable sutures (Johnson and Johnson, Düsseldorf, Germany).

Either Fast Blue (FB, Polysciences, Warrington, PA, USA) or modified double stranded Adeno-Associated Virus [19] expressing DsRed-Express (AAV-REx) were used to target neurons in the cortico- and rubrospinal tract. Double stranded Adeno-Associated virus (AAV) was chosen as a second tracer because it demonstrates superior and accelerated transduction *in vitro* and *in vivo* [21]. Shortly before onset of clinical scrapie (see, Tables S1, S2, S3 and S4), mice were anesthetized with Ketanest/Rompun, a cut was made on the level of cervical spinal cord and 1 µl of either 0.1% FB or AAV (1×10^9^ virus particles/ml) were injected into the cervical spinal cord with a 33-gauge Hamilton syringe over a period of 2 minutes. After inoculation, the wound was closed with Vicryl resorbable sutures (Johnson and Johnson, Düsseldorf, Germany).

### Sample preparation

The animals (see Tables S1, S2, S3 and S4) were sacrificed either at terminal stage of the disease, at the time point exactly matching the tracer injection or a week later – to co-localize REx, PrP^Sc^ or different markers. Mice were sacrificed using CO_2_ and immediately perfused transcardially with phosphate-buffered saline (PBS) followed with 4% paraformaldehyde in sodium phosphate buffer (pH 7.2). The samples for neuron quantification and immunohistochemistry with subsequent imaging using confocal microscopy were post-fixed in 4% paraformaldehyde for 1 hour at 4°C, incubated overnight with 20% sucrose in phosphate buffered saline (PBS) for cryo-protection at 4°C and frozen in Tissue Tek (Sakura, Zoeterwoude, The Netherlands) followed by performing 20 µm-thick cryo-sections. In parallel, the samples for ultramicroscopy were prepared as reported previously [18],[34]. Briefly, they were post-fixed in 4% paraformaldehyde for 1 hour at 4°C, washed 2 hours in PBS and subsequently in 30%, 50%, 70%, 80%, 96%, and 2 times in 100% alcohol at room temperature 10 hours each. After 1 h incubation in n-hexane (Sigma-Aldrich Chemie GmbH, Munich, Germany) the samples were incubated with Clearing Solution (CS, 1 part of benzyl alcohol and 2 parts of benzyl benzoate, both Sigma-Aldrich Chemie GmbH, Munich, Germany), 3–4 times 20–30 minutes each carefully avoiding air contact. The samples were incubated in CS at room temperature for further 14–18 hours immediately before imaging.

### Confocal microscopy

The tracer-positive neurons or immunohistochemistry in red nucleus (RN) or motor cortex (MC) were imaged on 20 µm-thick cryo-sections. The tracer-positive neurons were visualized on a Leica SP5 (Leica, Mannheim, Germany) laser-scanning confocal microscope using a 20×objective with numerical aperture (NA) of 0.7. Excitation wavelengths of 405 nm and 514 nm were used and the excited light was analyzed at 500 to 550 and 530 to 580 ranges for FB and AAV, respectively. This version of confocal microscope allows for storing exact laser and microscope specifications, which was done for standardizing the imaging and quantification of tracer-positive cells. The confocal stacks were taken to visualize approximately every µm of a given section.

### Ultramicroscopy

The home-built ultramicroscopy system was previously described [18]. Briefly, it included an Ar Ion Laser (Innova I-308, Coherent, Santa Clara, CA, USA) and an objective inverter (LSM Tech, Stewartstown, PA, USA) built into a commercial inverted microscope (Axiovert 200, Zeiss, Göttingen, Germany). A 10 mm-tall light sheet was created by focusing the beam with a cylindrical lens (focal length 5 cm, Newport, Irvine, CA, USA) and adjusted with a mirror (Thor Labs, Auburn, CA, USA). To focus the light sheet, the cylindrical lens was moved using a translation stage (Standa, Vilnius, Lithuania).

Epiplan 10×with 0.2 numerical aperture (NA) and LD Achroplan 20×with 0.4 NA objectives (Zeiss, Göttingen, Germany) were used in this work. They were placed 14 and 6 millimeters from a home-built 36×25 mm rectangular sample chamber, respectively. The specimen was affixed with Pattex acryl amide glue (Henkel, Düsseldorf, Germany) to a home-built glass rod. The sample was positioned with a stepper motor-controlled stage (Standa, Vilnius, Lithuania) with a feedback-memory system, XY accuracy of 0.1 µm and rotational accuracy of 0.1 degrees. The stepper motors were controlled by the software package LabView (National Instruments, Austin, TA, USA).

The emission light beam was let through the objective inverter (LSM Tech, Stewartstown, PA, USA), a dichroic mirror (DCLP 555, Chroma, Rockingham, VT, USA) and a band pass filter (HQ 607/75, Chroma, Rockingham, VT, USA) to remove any scattered excitation light from the laser. A back-illuminated electron multiplying charge-coupled device (EMCCD) camera with resolution of 512×512 pixels, 16 µm/pixel (Cascade II, Photometrics, Tucson AZ, USA) was used in this work. Laser illumination was synchronized with EMCCD camera exposure, as previously reported [18]. The exposure time for image acquisition was 500 ms per frame. Electronic output signal from the EMCCD camera was filtered and temporally readjusted using a function generator (Hameg, Mainhausen, Germany). Data acquisition and storage were administered by the software package MetaMorph 7.1 (Molecular Devices, Downingtown, PA, USA).

### Cell quantification and image analysis

The red nucleus (RN) is a center in the midbrain localized between anteroposterior (bregma) coordinates −4.2 and −3.3 [35]. The RN region was anatomically localized, and typically 35–40 serial sections were made through this area. The sections were imaged starting from the caudal part of the RN. We made confocal z-stacks on each of analyzed serial sections containing retrogradely labeled neurons to further quantify the labeled cells. The cell quantification was started upon the appearance of tracer-positive cells. Typically 10–12 of 20–25 µm-thick serial sections were analyzed starting from the initial labeled cell appearance. These sections covered the whole parvicellular and majority of magnocellular part of RN. Counting ceased upon the disappearance of labeled neurons. FB and REx both localize in the cytoplasm. Typically the time between tracer application and sacrificing the mice with subsequent sample analysis was quite long (up to 163 days), so the traced cells were not visible as a whole, as was shown elsewhere [36], but contained several irregular fluorescent objects together forming the cell shape with typical empty area in the place of nucleus. The cell shapes and sizes were defined by the NeuN immunostaining. Due to non-uniform fluorescence of FB and REx in the cytozol, we could not always apply automated software analysis for cell quantification, but quantified visually. For un-biased results we compared the data obtained with two independent researchers. The analysis of z-stacks enabled the reliable recognition of large pyramidal neurons with diameters between 12 and 25 µm, discrimination of the signal from high background (signal-to-background ratio of less than 1.5) and exclusion of artifacts. We considered the small fluorescent objects up to 2 to 3 µm in diameter not forming the cellular shapes as artifacts. These objects were observed in all brain areas, not only in the RN. A slightly enhanced density of these objects was observed in the i.n. inoculated Tga20 mice but not in any other mouse line, and it did not affect the cell count, especially shown by the co-localization and Region of Interest (ROI) fluorescence analyses. Additionally we confirmed the localization for the region of interest using *Wisteria floribunda* agglutinin (WFA) staining, a typical RN stain negative for neighboring tissues. Moreover, the tracer positive cells in RN were co-localized with NeuN and PGP9.5 neuronal markers. In order to evaluate the size parameters, we performed control quantification of neurons on z-stacks from a confocal microscope (see instrumentation) with NeuN-stained RN cryo-sections, which revealed approximately 10% difference between automated (done using two software packages, ImageJ and Volocity, see imaging and analysis of immunohistochemistry experiments) and manual analyses.

The large motor cortex (MC) is located between the anteroposterior (bregma) coordinates -1.3 and 2.4. The hindlimb part of the MC could be mapped by the microstimulation method caudally from the anteroposterior (bregma) coordinate −1 [37]. For the analysis of the confocal microscopy images, we took sixty 20 µm thick serial cryo-sections starting from bregma −1.7 to −1.5 according to the brain anatomy. We examined the sections until the number of tracer-positive neurons was approximately constant, and analyzed the first 13 sections, which covered an area of approximately 260 µm and quantified the labeled cells on every third section. Analysis of tracer-positive neurons in the MC was possible for animals traced with AAV-REx only. Two z-stacks for each side of all quantified MC sections were made on confocal microscope. In order to normalize the quantification, 5 sections for each mouse were observed. Z-projection of each stack was performed on the ImageJ software package (Psion Image, NIH, Bethesda, MD, USA). Z-projections for each side of the MC were combined in Adobe Photoshop (Adobe Systems, Munich, Germany) to obtain a complete picture of tracer-positive neurons on the given side of given section. The images were subsequently combined in one stack for each mouse. The quantification parameters (brightness threshold 85% and size threshold of 60–300 µm^2^) were normalized according to the results of REx co localization with NeuN-positive neurons in the MC of mock controls.

The ultramicroscopy stacks were analyzed in a 250 mm-thick area as reported previously [18]. Briefly, the obtained stacks were combined in one file and analyzed in ImageJ software with a brightness threshold of 80% fluorescence and size threshold of 80–150 µm^2^. This area was anatomically localized to the same region. Furthermore, we demonstrated previously that the ultramicroscopy technique allowed us to analyze and perform three-dimensional reconstruction of areas up to 4.2 mm long. The tracer- positive cells formed an area about 800–900 µm long with a relatively constant density of labeled cells starting from the bregma coordinate −1.2 to 1.5. These properties did not differ between mock and prion infected animals, but the cell density was dramatically reduced [18]. All these properties enabled a reliable, uniform localization and normalization of the cell counting between single probes as well as between ultramicroscopy and confocal imaging.

### Toluidine Blue staining

Toluidine Blue stains the myelin sheath and therefore can be used to visualize the neuronal projections in the spinal cord. The 5 µm thick paraffin spinal cord sections of wild-type either mock, i.c. or i.n. prion inoculated mice sacrificed at the terminal stage of the disease were taken for the experiment. After deparaffinization and staining in 0.1% Toluidine Blue in 1% NaCl, the sections were imaged with a light microscope (Leica SP5, Mannheim, Germany).

### Immunohistochemistry

20 µm cryo-sections were used for immunohistochemistry. Applied antibodies used were: mouse monoclonal NeuN (33 µg/ml, Millipore, Schwalbach, Germany); goat polyclonal Rab7 (6.7 µg/ml, Santa-Cruz, Heidelberg, Germany); mouse monoclonal p150^GLUED^ dynactin (8.3 µg/ml, BD Transduction, Heidelberg, Germany) and goat polyclonal Notch (6.7 µg/ml, Santa-Cruz, Heidelberg, Germany).

PrP^Sc^ was visualized with mouse monoclonal ICSM35 antibody (11 µg/ml, D-Gen, London, UK). In order to remove PrP^C^, cryo-sections were treated 10 min with 99.9% formic acid.

Secondary antibodies used were: for NeuN – rabbit anti-mouse IgG coupled with Alexa 546 (1 µg/ml, Invitrogen, Karlsuhe, Germany); for Rab7 and Notch – biotin-SP-conjugated AffiniPure rabbit anti-goat IgG (1∶100, Dianova, Hamburg, Germany) combined with Cy5-conjugated streptavidin (1∶100, Dianova) and for p150^GLUED^ dynactin – biotin-SP-conjugated AffiniPure rabbit anti-mouse IgG (1∶100, Dianova, Hamburg, Germany) followed by Cy5-conjugated streptavidin.

### Imaging and analysis of immunohistochemistry experiments

Quantification of NeuN-positive neurons was performed on Volocity (Improvision, Coventry, England) and ImageJ (Psion Image, NIH, Bethesda, MD, USA) software packages upon application of a size threshold of 2500 µm^3^ (18 µm approximate cell diameter) for the caudal RN part and 1000 µm^3^ (14 µm approximate cell diameter) – for the middle and the cranial RN part. For both RN parts the fluorescence threshold was taken as 80% of maximal fluorescence. All co localization analyses were performed with Volocity software (Improvision, Coventry, England) using z-stacks done on 20 µm-thick cryo-sections.

Analysis of the fluorescence in the region of interest (ROI) was made with ImageJ software (Psion Image, NIH, Bethesda, MD, USA) on z-stacks previously obtained on cryo-sections stained with anti-Rab7 antibodies using confocal microscope. The regions of interest were chosen according to the REx-positive cell debris. The mean fluorescence values were measured in exactly the same ROI for appropriate channels throughout the z-stacks. Subsequently, obtained data was transferred to the Excel program (Microsoft Deutschland GmbH Unterschleiβheim, Deutschland), summarized, and the percentage of the FB to NeuN, REx to NeuN, or Rab7 to the REx fluorescence in the same ROI was quantified for each cell. These data were transferred to either Graph Pad Prism 4.0 (GraphPad Software Inc., La Jolla, CA, USA) or OriginPro (Origin Lab Corporation, Northampton MA, USA) software packages for making the graphs and statistical analysis.

For the co localization analyses the concatenated confocal z-stacks were done on ImageJ (Psion Image, NIH, Bethesda, MD, USA) and subsequently analysed on Volocity software (Improvision, Coventry, England). The co-localization was shown in the yellow color throughout the whole manuscript.

The quantification of Ubiquitin- and Notch-positive cells in the MC was done with ImageJ software (Psion Image, NIH, Bethesda, MD, USA) on confocal images done on 3 to 5 different wt mice at 145 dpi after i.n. prion infection. The size thresholds of 80–500 µm^2^ and 30–300 µm^2^ were taken for ubiquitin and Notch quantifications, respectively. The 80% fluorescence intensity threshold was taken for both of them.

### Statistics

Unpaired t-tests as well as survival analyses were done using Graph Pad Prism 4.0 software (GraphPad Software Inc., La Jolla, CA, USA). N indicates the number of mice or samples in the experimental set. All error indications are standard deviations.

## Supporting Information

Figure S1 Neuropathology in the RN of wt mice infected with prions into the right sciatic nerve. Paraffin brain sections were prepared from wt mice immediately before onset of clinical prion disease (145 dpi) and stained with an antibody against glial fibrillary acidic protein (GFAP), a marker for activated astrocytes. Neuropathology is comparable on the sides contralateral and ipsilateral to prion challenge site. Scale bar: 100 µm.
(0.12 MB PDF)

Figure S2 The number of Fast Blue (FB)-positive cells (green), which co-localize with NeuN positive cells (red), is significantly different between contralateral and ipsilateral sides of the red nucleus immediately before the onset of clinical prion disease. The wild type mice were challenged with prions in the right sciatic nerve (i. n.) and the FB tracer was injected at 145 days post inoculation into the spinal cord. The white boxes are areas that were shown for co-localization analysis on the Figure 3A. Co-localization of FB and NeuN is shown with yellow. Scale bars, 100 µm.
(0.31 MB PDF)

Figure S3 The Regions of Interest (ROI) were defined according to the REx-positive cells (white lines) for the subsequent fluorescence analysis. (A) ROI on Fast Blue and NeuN profiles (see, Figure 3A). (B) ROI on REx and Rab7 profiles (see, Figure 6A and B). Scale bars, 50 µm.
(0.29 MB PDF)

Figure S4 p150GLUED staining reveals diminished immunoreactivity in the red nucleus of prion-challenged wt mice immediately before the onset of prion disease (at 145 dpi upon i.n. prion challenge) as compared to the mock control. NC - negative control without primary antibody. Scale bar: 100 µm.
(0.04 MB PDF)

Figure S5 Dynamitin immunoreactivity in the motor cortex does not differ between the mock- and prion-inoculated wt mice immediately before the onset of prion disease (at 145 dpi upon i.n. prion challenge). NC - negative control without primary antibody. Scale bar: 100 µm.
(0.06 MB PDF)

Figure S6 Toluidine Blue staining of wild type mouse cervical spinal cord in intracerebrally (i.c.) and intranervously (i.n.) challenged animals as compared to mock control. Different degrees of axonal swellings (upper - minimal to no swelling and lower - more swelling) is visible in the samples from different prion-infected and control mice. The stainings were done on paraffin sections from mock and prion challenged mice sacrificed at the terminal stage of the disease. Scale bar: 100 µm.
(0.68 MB PDF)

Table S1 Fast Blue-positive (FB+) neurons in red nucleus (RN) upon intracerebral (i.c.) prion challenge with 1% RML mouse prions.
(0.01 MB PDF)

Table S2 FB-positive neurons (FB+) in the red nucleus (RN) of TG(SHaPrP) transgenic mice upon i.c. and i.n. prion challenge with 1% Sc237 hamster prions.
(0.01 MB PDF)

Table S3 FB-positive neurons (FB+) in red nucleus (RN) after prion challenge into the right sciatic nerve (i.n.) with 1% RML mouse prions.
(0.01 MB PDF)

Table S4 DsRed-Express positive (REx+) neurons in the red nucleus (RN) upon i.n. challenge with 1% RML mouse prions.
(0.01 MB PDF)

Table S5 Tracer-positive and NeuN-positive neurons in rednucleus (RN) of wt mice after prion challenge into the right sciaticnerve (i.n.).
(0.01 MB PDF)
