# Supplementary figures and images for: Impaired Axonal Transport in Motor Neurons Correlates with Clinical Prion Disease
Source: PLoS Pathog. 2009 Aug 21;5(8):e1000558. doi: 10.1371/journal.ppat.1000558 (PMC2723930; doi:10.1371/journal.ppat.1000558)

contralateral

ipsilateral

mock

prions i.n.

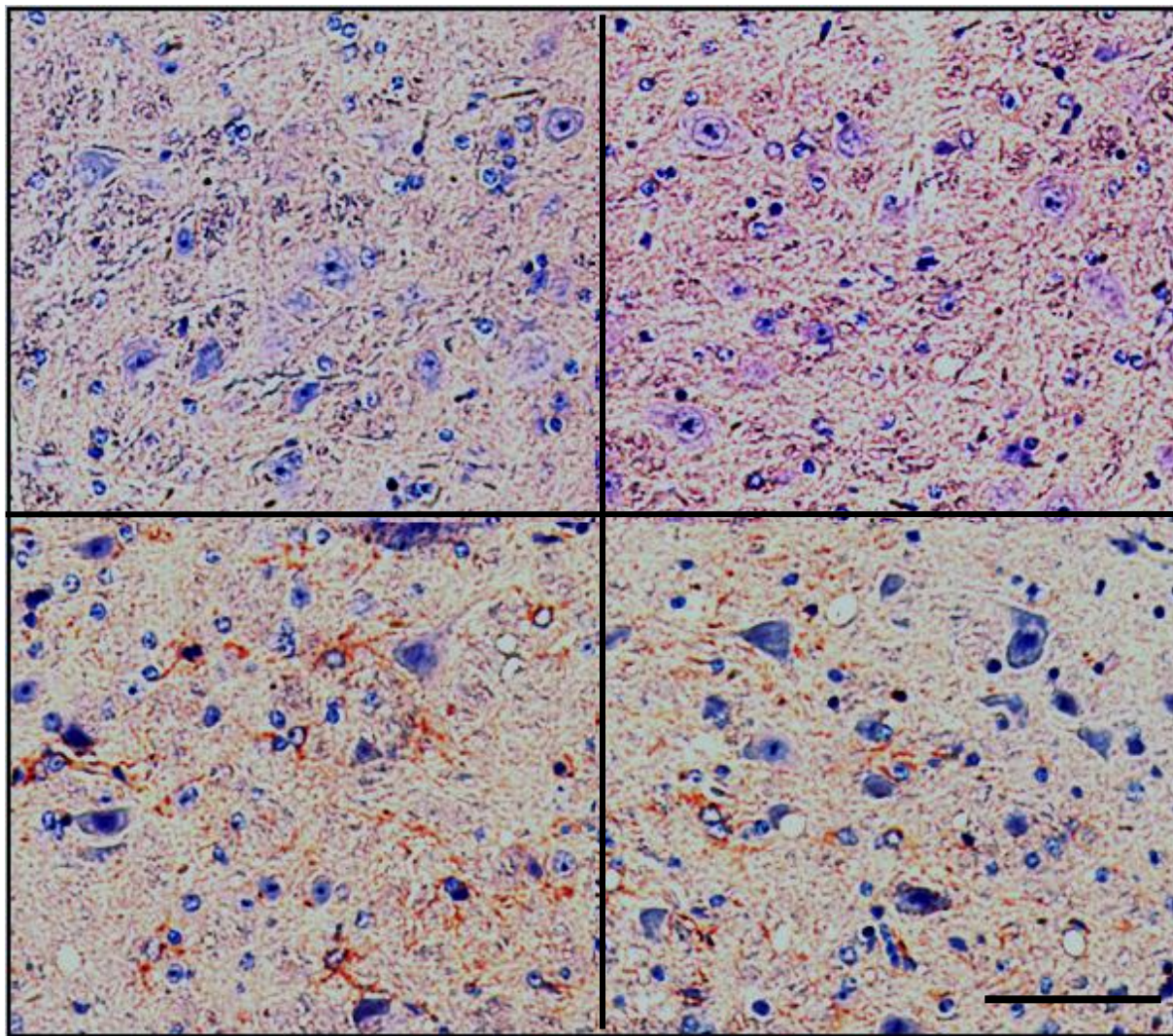

Supplement: Figure S1 — Neuropathology in the RN of wt mice infected with prions into the right sciatic nerve. Paraffin brain sections were prepared from wt mice immediately before onset of clinical prion disease (145 dpi) and stained with an antibody against glial fibrillary acidic protein (GFAP), a marker for activated astrocytes. Neuropathology is comparable on the sides contralateral and ipsilateral to prion challenge site. Scale bar: 100 µm. (0.12 MB PDF) [file ppat.1000558.s001.pdf]

Fast Blue

NeuN

Merge

prions i.n., contralateral

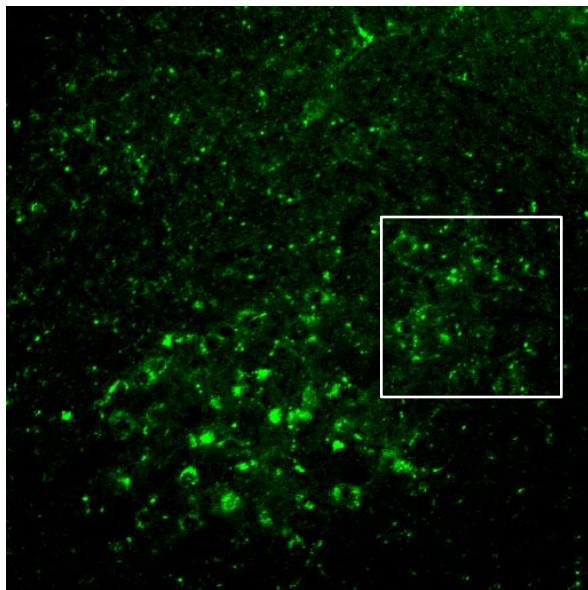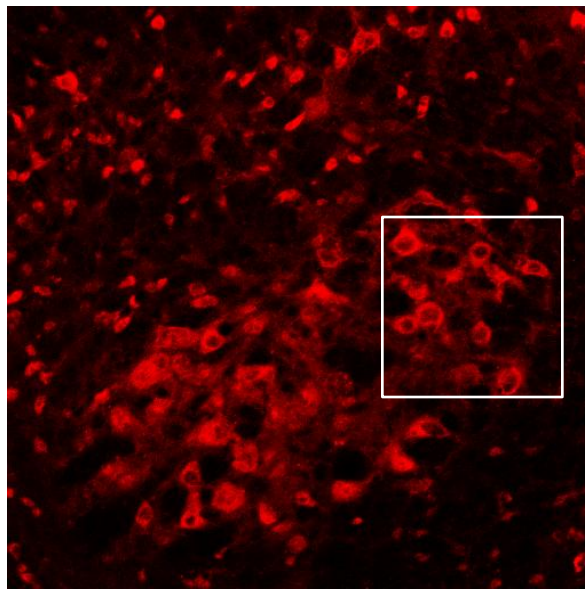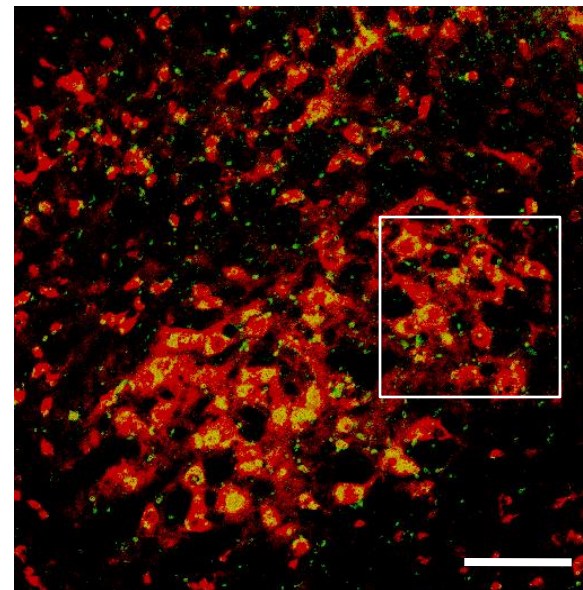

prions i.n., ipsilateral

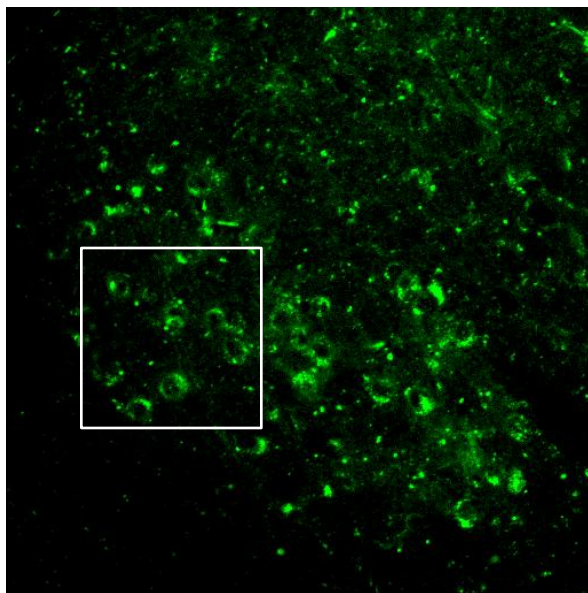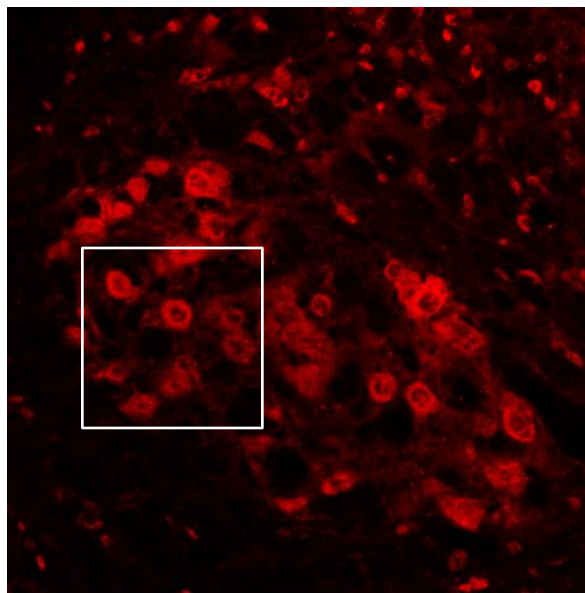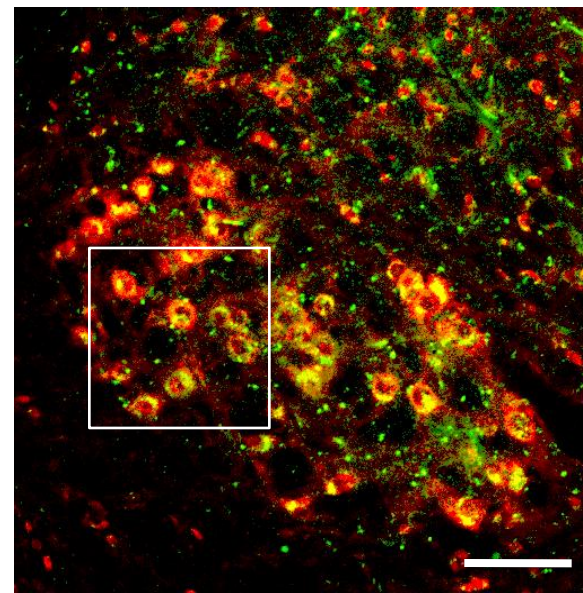

Supplement: Figure S2 — The number of Fast Blue (FB)-positive cells (green), which co-localize with NeuN positive cells (red), is significantly different between contralateral and ipsilateral sides of the red nucleus immediately before the onset of clinical prion disease. The wild type mice were challenged with prions in the right sciatic nerve (i. n.) and the FB tracer was injected at 145 days post inoculation into the spinal cord. The white boxes are areas that were shown for co-localization analysis on the Figure 3A. Co-localization of FB and NeuN is shown with yellow. Scale bars, 100 µm. (0.31 MB PDF) [file ppat.1000558.s002.pdf]

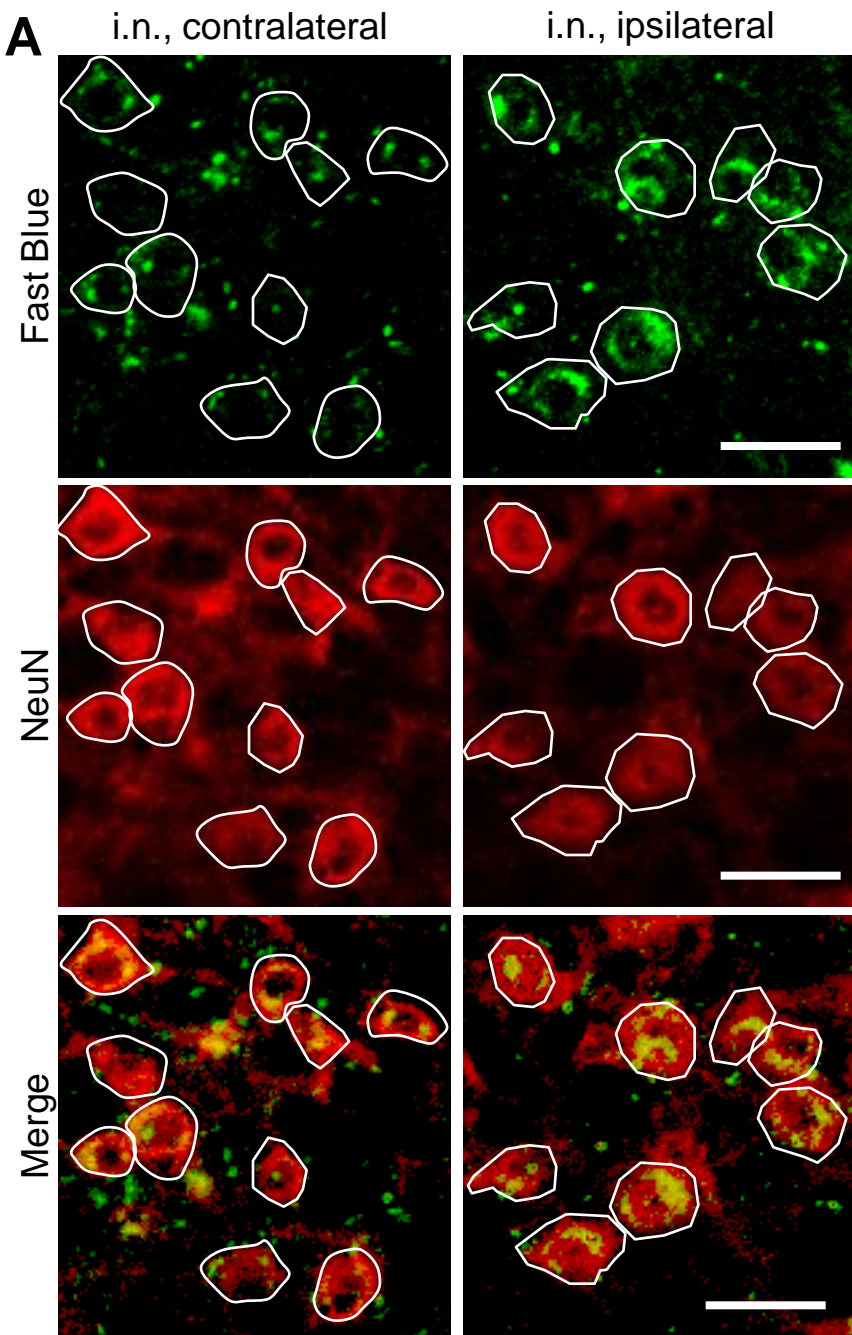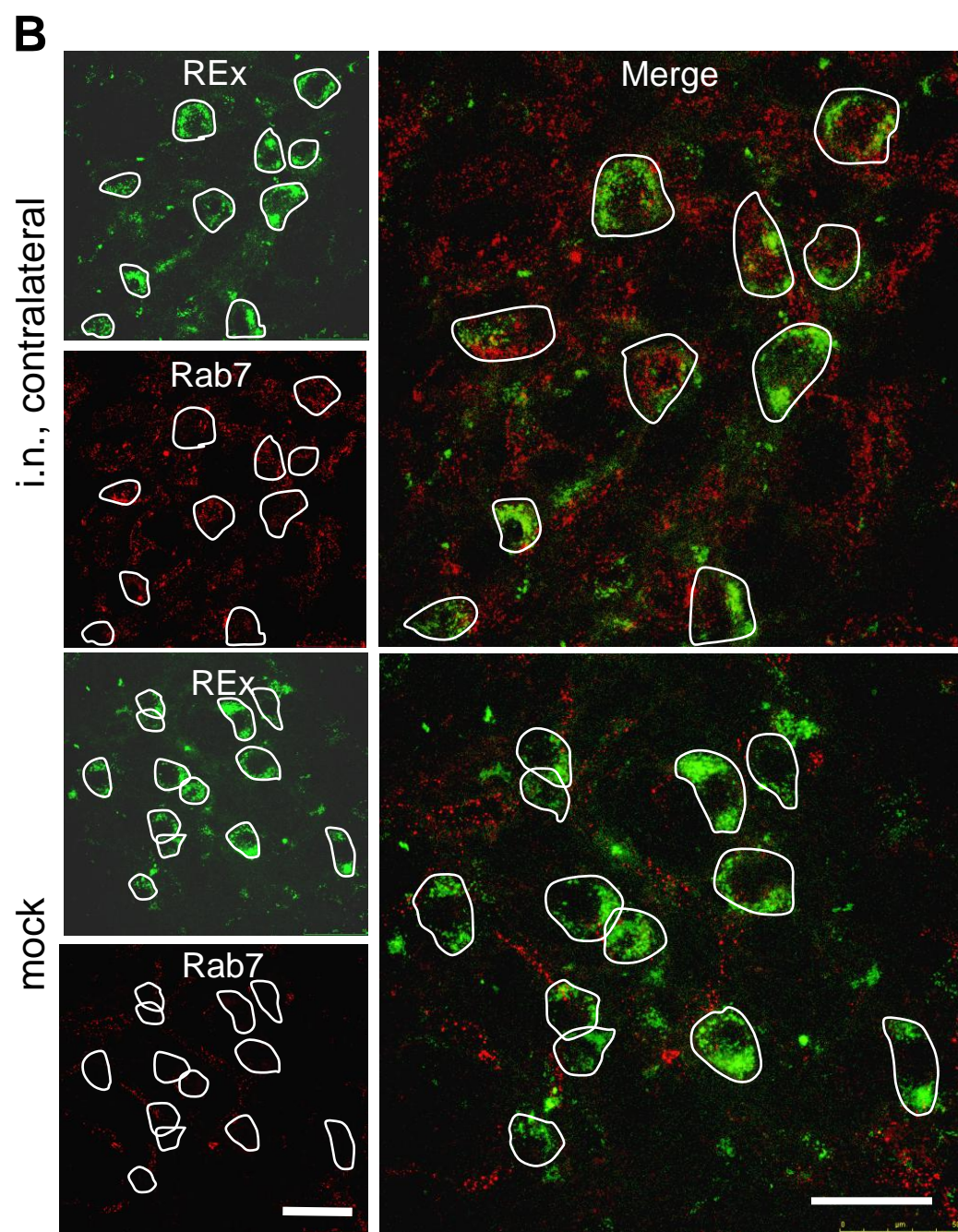

Supplement: Figure S3 — The Regions of Interest (ROI) were defined according to the REx-positive cells (white lines) for the subsequent fluorescence analysis. (A) ROI on Fast Blue and NeuN profiles (see, Figure 3A). (B) ROI on REx and Rab7 profiles (see, Figure 6A and B). Scale bars, 50 µm. (0.29 MB PDF) [file ppat.1000558.s003.pdf]

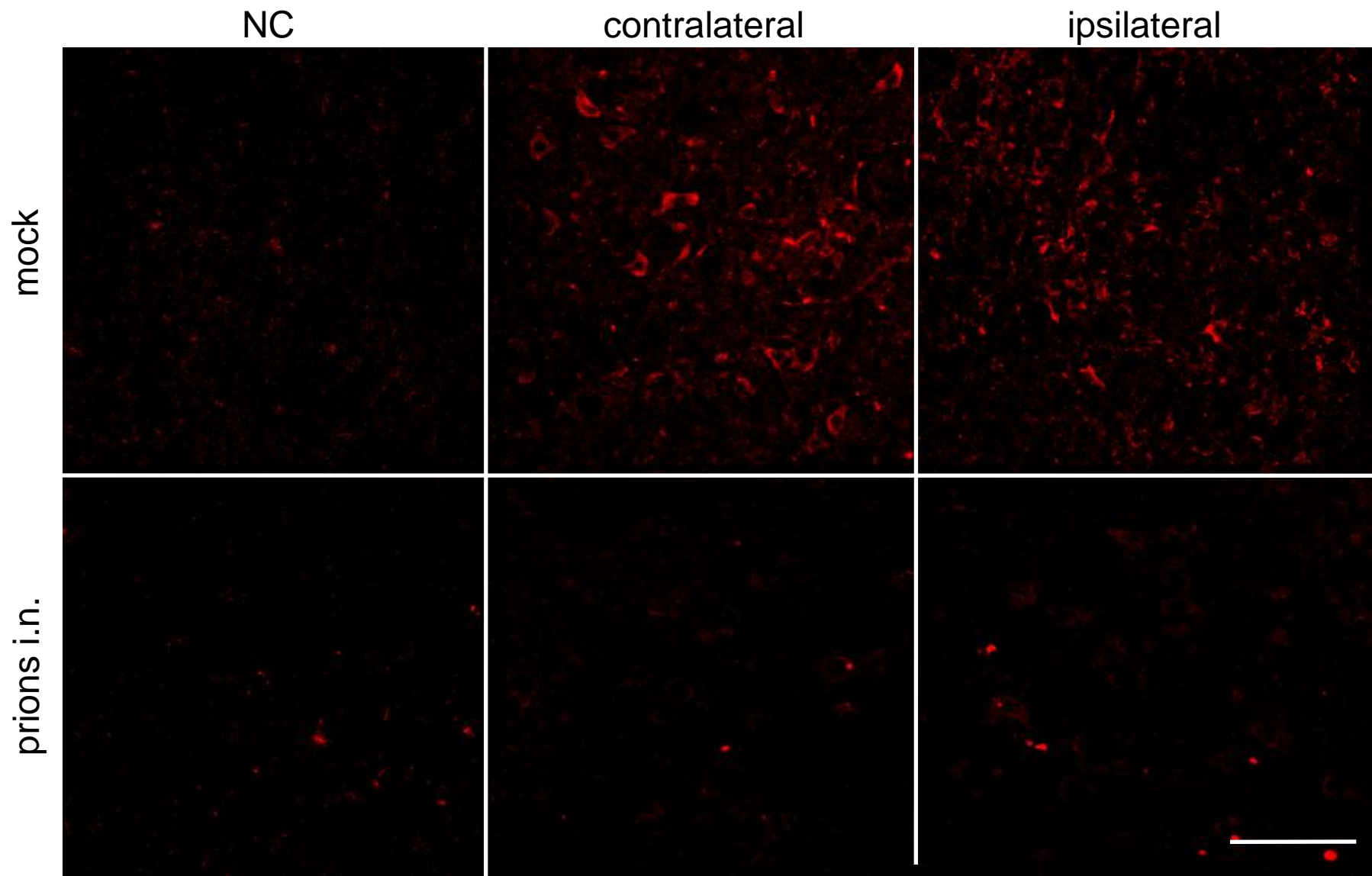

Supplement: Figure S4 — p150GLUED staining reveals diminished immunoreactivity in the red nucleus of prion-challenged wt mice immediately before the onset of prion disease (at 145 dpi upon i.n. prion challenge) as compared to the mock control. NC - negative control without primary antibody. Scale bar: 100 µm. (0.04 MB PDF) [file ppat.1000558.s004.pdf]

NC

contralateral

ipsilateral

mock

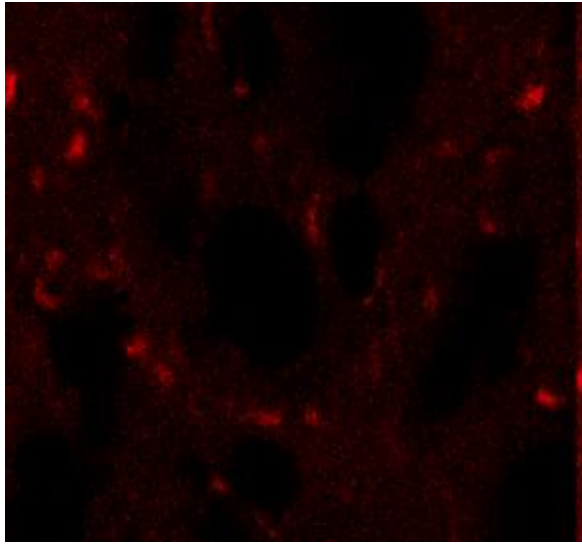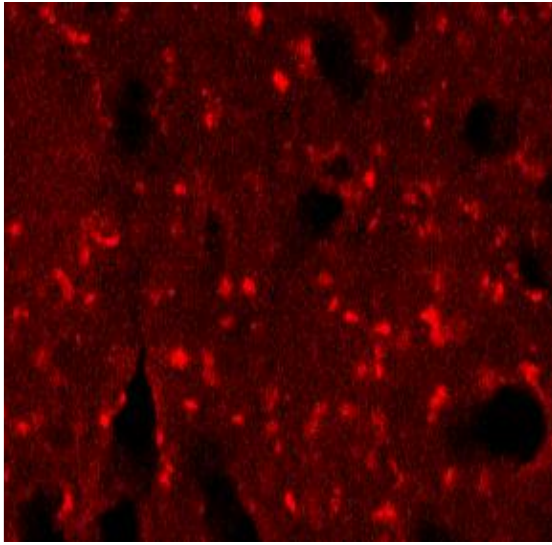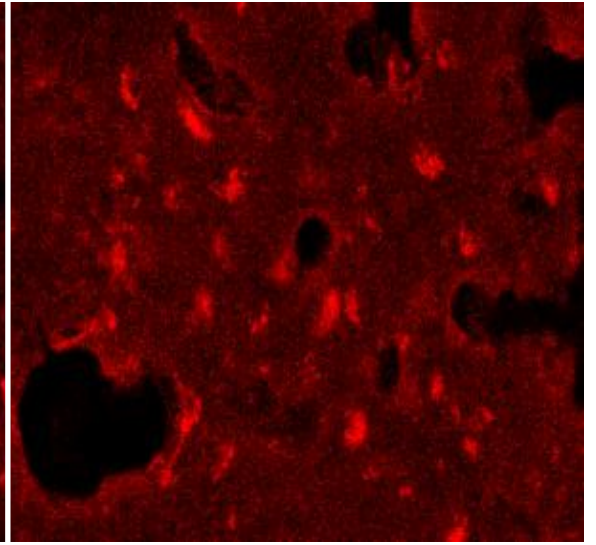

prions i.n.

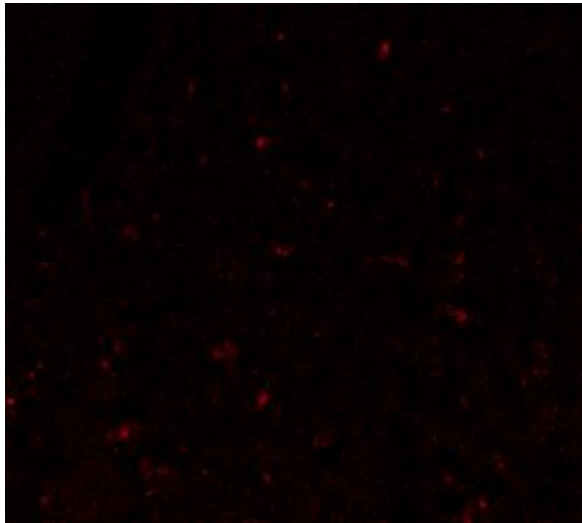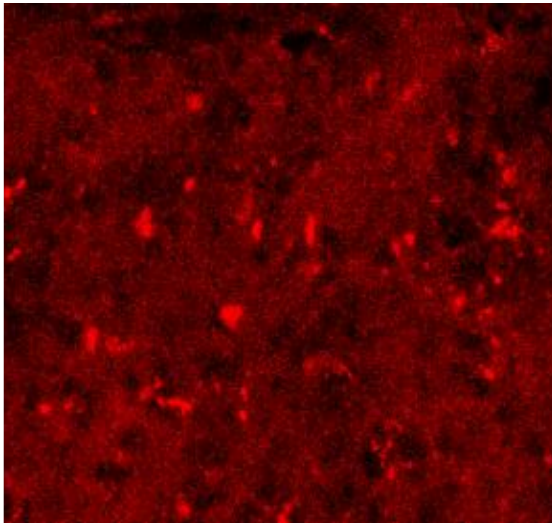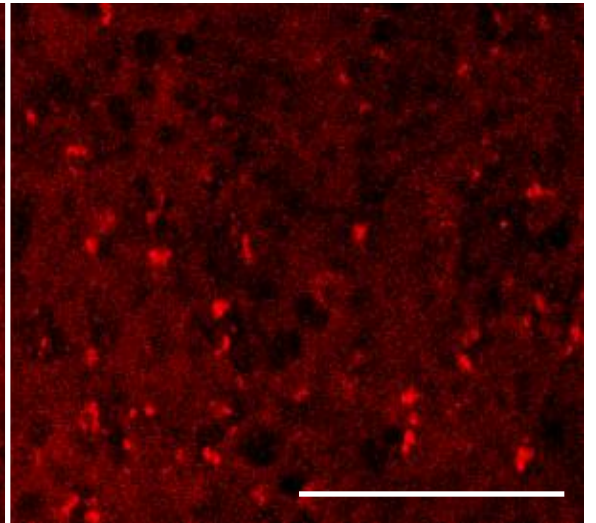

Supplement: Figure S5 — Dynamitin immunoreactivity in the motor cortex does not differ between the mock- and prion-inoculated wt mice immediately before the onset of prion disease (at 145 dpi upon i.n. prion challenge). NC - negative control without primary antibody. Scale bar: 100 µm. (0.06 MB PDF) [file ppat.1000558.s005.pdf]

mock

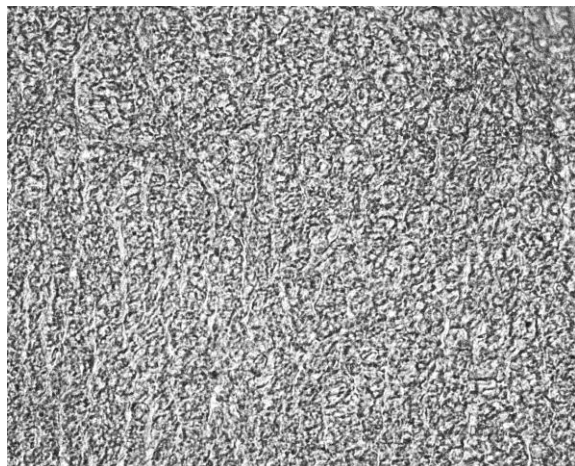

i.n.

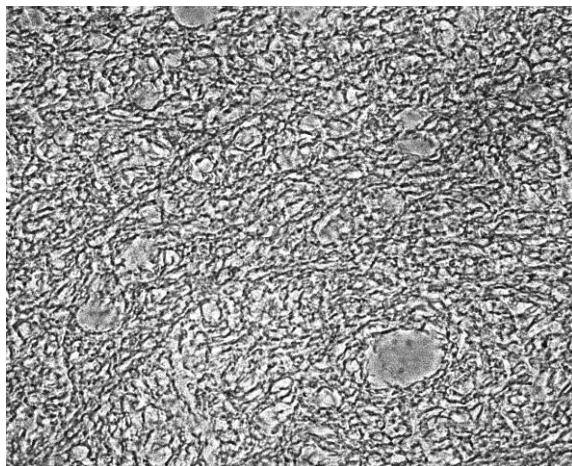

i.c.

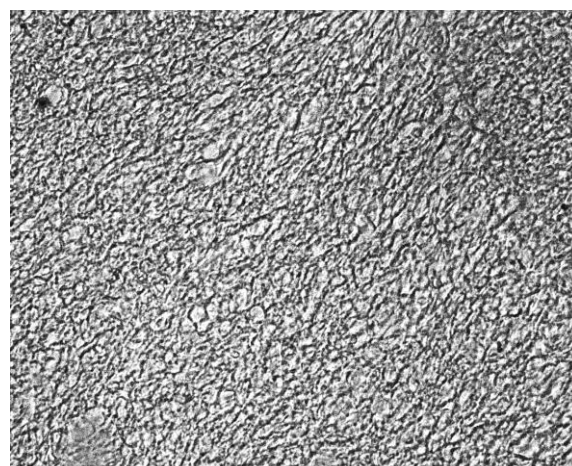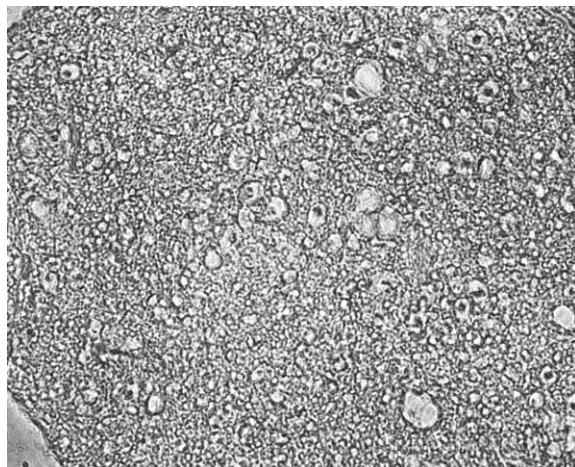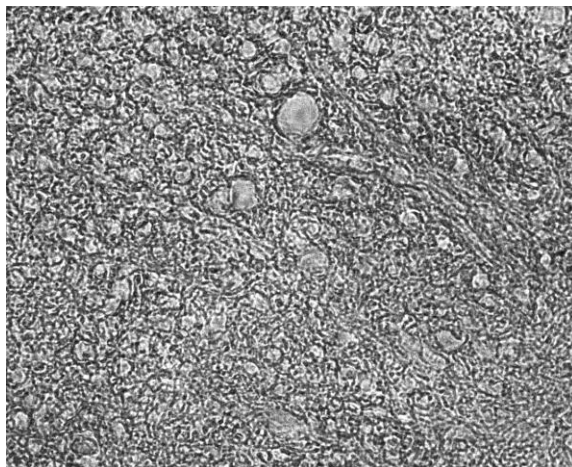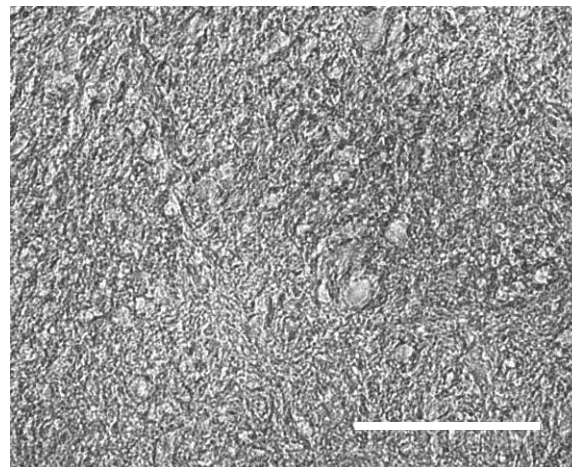

Supplement: Figure S6 — Toluidine Blue staining of wild type mouse cervical spinal cord in intracerebrally (i.c.) and intranervously (i.n.) challenged animals as compared to mock control. Different degrees of axonal swellings (upper - minimal to no swelling and lower - more swelling) is visible in the samples from different prion-infected and control mice. The stainings were done on paraffin sections from mock and prion challenged mice sacrificed at the terminal stage of the disease. Scale bar: 100 µm. (0.68 MB PDF) [file ppat.1000558.s006.pdf]
