# Supplementary material for: Impaired Axonal Transport in Motor Neurons Correlates with Clinical Prion Disease
Source: PLoS Pathog. 2009 Aug 21;5(8):e1000558. doi: 10.1371/journal.ppat.1000558 (PMC2723930; doi:10.1371/journal.ppat.1000558)
Supplement: Table S1 — Fast Blue-positive (FB+) neurons in red nucleus (RN) upon intracerebral (i.c.) prion challenge with 1% RML mouse prions. (0.01 MB PDF) [file ppat.1000558.s007.pdf]

Table S1. Fast Blue-positive (FB+) neurons in red nucleus (RN) upon intracerebral (i.c.) prion challenge with 1% RML mouse prions.

Mouse line	wt (C57Bl/6)**		Tga20		C4/C4		*$Prnp^{0/+}$*			
Inoculum (i.c. route)	1% mock	1% RML	1% mock	1% RML	1% mock	1% RML	1% mock	1% RML	1% RML	1% RML
FB+ neurons in RN	206±19	117±6	232±14	129±13	205±5	143±6	231±7	143±22	127±10	108±15
% to Mock controls	100±9	57±6	100±6	55±6	100±2	70±3	100±3	62±10	55±5	51±8
Tracer inoculation, dpi*	131	131	55	55	163	163	270	270	330	370
Scrapie onset, dpi*	–	146±2	–	59±1	–	203±3	–		322±2	
Terminal disease, dpi*	–	159±4	–	63±3	–	237±3	–		410±28	
N/N0	0/5	4/4	0/4	4/4	0/3	8/8	0/2	3/3	3/3	3/3

* dpi – days post inoculation; **wt – wild type; all values given are: mean value ± standard deviation of the mean.
