# Supplementary material for: Impaired Axonal Transport in Motor Neurons Correlates with Clinical Prion Disease
Source: PLoS Pathog. 2009 Aug 21;5(8):e1000558. doi: 10.1371/journal.ppat.1000558 (PMC2723930; doi:10.1371/journal.ppat.1000558)
Supplement: Table S2 — FB-positive neurons (FB+) in the red nucleus (RN) of TG(SHaPrP) transgenic mice upon i.c. and i.n. prion challenge with 1% Sc237 hamster prions. (0.01 MB PDF) [file ppat.1000558.s008.pdf]

Table S2. FB-positive neurons (FB+) in the red nucleus (RN) of TG(SHaPrP) transgenic mice upon i.c. and i.n. prion challenge with 1% Sc237 hamster prions.

Mouse line	TG(SHaPrP)				
route	i.c.	i.c.	i.n.	i.n.	i.n.
Inoculum	1% mock	1% RML	1% mock	1% RML	1% RML
Side of RN		contra*		contra*	ipsi**
Tracer-positive neurons in RN	171±15	125±9	171±15	97±14	170±7
Per cent to Mock controls	100±9	73±5	100±9	57±4	100±4
Tracer inoculation, dpi	53	53	67	67	
Scrapie onset, dpi		53±1		73±8	
Terminal disease, dpi		55±2		75±8	
N/N0	0/2	6/6	0/5	5/5	

*contra – contralateral to the inoculation in the right sciatic nerve; **ipsi – ipsilateral to the inoculation in the right sciatic nerve; all values given are: mean value ± standard deviation of the mean.
