# Supplementary material for: Impaired Axonal Transport in Motor Neurons Correlates with Clinical Prion Disease
Source: PLoS Pathog. 2009 Aug 21;5(8):e1000558. doi: 10.1371/journal.ppat.1000558 (PMC2723930; doi:10.1371/journal.ppat.1000558)
Supplement: Table S3 — FB-positive neurons (FB+) in red nucleus (RN) after prion challenge into the right sciatic nerve (i.n.) with 1% RML mouse prions. (0.01 MB PDF) [file ppat.1000558.s009.pdf]

Table S3. FB-positive neurons (FB+) in red nucleus (RN) after prion challenge into the right sciatic nerve (i.n.) with 1% RML mouse prions.

Mouse line	wt			Tga20			C4/C4		
Inoculum (i.n. route)	1% mock	1% RML	1% RML	1% mock	1% RML	1% RML	1% mock	1% RML	1% RML
RN side		contra*	ipsi**		contra*	ipsi**		contra*	ipsi**
FB+ neurons in RN	171±15	97±14	170±7	232±14	129±13	246±19	206±5	138±18	210±20
Per cent to Mock controls	100±5	56±4	101±1	100±6	63±5	103±8	100±3	67±9	102±10
Tracer inoculation, dpi	145	145		54	54		171	171	
Scrapie onset, dpi	–	149±5		–	64±6		–	184±11	
Terminal disease, dpi	–	176±3		–	72±4		–	204±18	
N/N0	0/3	4/4		0/4	4/4		0/2	4/4	

*contra – contralateral to the inoculation in the right sciatic nerve; **ipsi – ipsilateral to the inoculation in the right sciatic nerve; all values given are: mean value ± standard deviation of the mean.
