# Supplementary material for: Impaired Axonal Transport in Motor Neurons Correlates with Clinical Prion Disease
Source: PLoS Pathog. 2009 Aug 21;5(8):e1000558. doi: 10.1371/journal.ppat.1000558 (PMC2723930; doi:10.1371/journal.ppat.1000558)
Supplement: Table S4 — DsRed-Express positive (REx+) neurons in the red nucleus (RN) upon i.n. challenge with 1% RML mouse prions. (0.01 MB PDF) [file ppat.1000558.s010.pdf]

**Table S4. DsRed-Express positive (REx+) neurons in the red nucleus (RN) upon i.n. challenge with 1% RML mouse prions.**

| Mouse line | Wt* (C57Bl/6) | | | | | Tga20 | | |
|---|---|---|---|---|---|---|---|---|
| Inoculum (i.n. route) | 1% mock | 1% RML | 1% RML | 1% RML | 1% RML | 1% mock | 1% RML | 1% RML |
| Side of RN | | contra** | ipsi*** | contra** | ipsi*** | | contra** | ipsi*** |
| Tracer-positive neurons in RN | 205±19 | 182±3 | 213±11 | 101±3 | 201±30 | 236±11 | 128±6 | 196±16 |
| Per cent to Mock controls | 100±9 | 90±6 | 103±5 | 61±7 | 98±15 | 100±5 | 51±2 | 86±3 |
| Tracer inoculation, dpi | 145 | 82 | | 145 | | 54 | 54 | |
| Scrapie onset, dpi | – | 149±5 | | 149±5 | | – | 64±6 | |
| Terminal disease, dpi | – | 176±3 | | 176±3 | | – | 72±4 | |
| N/N0 | 0/3 | 4/4 | | 2/2 | | 0/2 | 6/6 | |

*wt – wild type; **contra – contralateral to the inoculation in the right sciatic nerve; ***ipsi – ipsilateral to the inoculation in the right sciatic nerve; all values given are: mean value ± standard deviation of the mean.
