# Supplementary material for: Impaired Axonal Transport in Motor Neurons Correlates with Clinical Prion Disease
Source: PLoS Pathog. 2009 Aug 21;5(8):e1000558. doi: 10.1371/journal.ppat.1000558 (PMC2723930; doi:10.1371/journal.ppat.1000558)
Supplement: Table S5 — Tracer-positive and NeuN-positive neurons in rednucleus (RN) of wt mice after prion challenge into the right sciaticnerve (i.n.). (0.01 MB PDF) [file ppat.1000558.s011.pdf]

Table S5. Tracer-positive and NeuN-positive neurons in red nucleus (RN) of wt mice after prion challenge into the right sciatic nerve (i.n.).

	Tracer-positive			NeuN-positive		
Inoculum (i.n. route)	1% mock	1% RML	1% RML	1% mock	1% RML	1% RML
RN side		contra*	ipsi**		contra*	ipsi**
Quantified neurons in RN	199±7	99±11	196±15	221±10	212±6	210±10
Per cent to Mock ref.***	97±3	48±5	95±7	108±3	103±3	102±5
Tracer inoculation, dpi	145	145		145	145	
Animal sacrificed at dpi§	152	152		152	152	
Scrapie onset, dpi§		149±5			149±5	
Terminal disease, dpi§		176±3			176±3	
N	3	4		3	4	

*contra – contralateral to the inoculation in the right sciatic nerve; **ipsi – ipsilateral to the inoculation in the right sciatic nerve; *** -- Mock ref.: number of REx+ neurons quantified in wt mock control mice (Table S4). § -- values taken from previous experiments, see Tables S2 and S4. All values given are: mean value ± standard deviation of the mean.
